# Psychopathology of psychiatric patients presenting autoantibodies against neuroglial antigens

**DOI:** 10.3389/fpsyt.2022.945549

**Published:** 2022-11-10

**Authors:** Insa Maria Grenzer, Aaron Levin Juhl, Bianca Teegen, Dirk Fitzner, Jens Wiltfang, Niels Hansen

**Affiliations:** ^1^Department of Psychiatry and Psychotherapy, University Medical Center Göttingen, Göttingen, Germany; ^2^Translational Psychoneuroscience, University Medical Center Göttingen, Göttingen, Germany; ^3^Clinical Immunological Laboratory Prof. Stöcker, Groß Grönau, Germany; ^4^Department of Neurology, University Medical Center Göttingen, Göttingen, Germany; ^5^German Center for Neurodegenerative Diseases (DZNE), Göttingen, Germany; ^6^Neurosciences and Signaling Group, Department of Medical Sciences, Institute of Biomedicine, University of Aveiro, Aveiro, Portugal

**Keywords:** psychopathology, neural autoantibody, autoimmunity, psychiatry, HiTOP classification, AMDP system

## Abstract

**Background:**

Autoantibody-mediated psychiatric disorder is often difficult to diagnose as the clinical features of psychiatric disorder associated with neural autoantibodies are often similar. Thus, it is of major relevance to investigate whether psychopathology can differentiate between both disease entities as a biomarker and help us in searching for specific autoantibodies associated with psychiatric symptoms.

**Methods:**

We enrolled 154 patients of the Department of Psychiatry and Psychotherapy of the University Medical Center Göttingen with psychopathology data and retrospectively evaluated their patient records using the classification systems AMDP (Arbeitsgemeinschaft für Methodik und Dokumentation in der Psychiatrie) and HiTOP (Hierarchical Taxonomy of Psychopathology).

**Results:**

We identified 35 psychiatric patients revealing autoantibodies in their serum and/or cerebrospinal fluid (CSF) and 119 with no autoantibodies. Relying on the AMDP system, many more psychiatric patients with serum autoantibodies (51%) had problems with orientation than those without autoantibodies (32%) (*p* < 0.05). Furthermore, fewer psychiatric patients with serum autoantibodies exhibited a blunted affect (11.4 vs. 32.8%, *p* < 0.01) and affective rigidity (20 vs. 45%, *p* < 0.01). In particular, psychiatric patients presenting CSF autoantibodies (indicating an autoimmune symptomatic basis) experience more loss of vitality (5%) than those without autoantibodies (0%) (*p* < 0.05). Another interesting finding is that according to the AMDP classification, a manic syndrome is much more frequent in autoantibody-positive (8.6%) than autoantibody-negative psychiatric patients (0.8%) (*p* < 0.05). Another aspect is the more frequent occurrence of attention and memory deficits in patients with autoantibodies against intracellular targets compared with targets on the membrane surface.

**Conclusion:**

Our findings indicate that neural autoantibodies in psychiatric patients could indicate a phenotype more often characterized by a manic syndrome, orientation disturbances within the cognitive spectrum, and fewer affect disturbances characterized by less blunted affect and not as seriously impaired feelings of vitality compared to controls. The novelty of our approach is the extensive autoantibody tests for various psychiatric syndromes in combination with a profound psychometric measurement with two different scales.

## Introduction

The quantification and diversity of psychopathology in neural autoantibody-associated psychiatric syndromes is an under-investigated topic in immunopsychiatry that deserves more attention since psychopathology may be an early important biomarker for diagnosing neural autoantibodies in psychiatric disorders. Neural autoantibodies associated psychiatric syndromes might be an initial manifestation of autoimmune encephalitis on the one hand. On the other hand, neural autoantibody-associated psychiatric disease might not fulfill autoimmune-encephalitis criteria. Autoimmune encephalitis is a designation that relies on specific criteria ranging from clinical features, like psychiatric symptoms and memory impairment, to diagnostic indices of central nervous system (CNS) inflammation derived from cerebrospinal fluid (CSF) analysis, neuroimaging, and electroencephalography (1). There are sometimes no clear indications of autoimmune encephalitis, although neural autoantibodies are associated with psychiatric syndromes. The distinction is therapeutically highly relevant, as there are clear therapeutic guidelines for autoimmune encephalitis or encephalopathy (1–3) where isolated serum autoantibodies have less clear meaning and a therapeutic intervention is limited to individual therapeutic healing attempts. In such cases, a consortium called “Cerebrospinal Fluid Analysis in Psychiatry” (4) developed additional clinical criteria such as a dynamic psychopathology (5) or catatonia (6) to diagnose an autoimmune basis of psychiatric syndrome presentation. These additional parameters have not been evaluated in large sample cohorts as biomarkers and therefore require validation in randomized large-scale trials. We are not yet aware of any novel early biomarkers that would improve such patients' diagnosis and therapy. The patient's phenotypic appearance is often an early hint implying the direction the diagnostic approach should take. Thus, psychopathological factors may eventually prove to be biomarkers that are better at distinguishing between patients who may possess neuronal autoantibodies than those who do not. This assumption is based on recent studies that show that specific psychopathological features are associated with neural autoantibodies, such as anti-adaptor-related protein complex 3, beta 2 subunit (AP3B2) autoantibodies in conjunction with persecutory delusions, or anti-tryptophan 2,3-dioxygenase (TDO2) antibodies (7) together with hallucinations in patients or N-methyl-D-aspartate receptor (NMDAR) antibodies in patients with a common coexisting depressive and psychotic symptomatology (8). Thus, the primary goal of the study is to assess the psychopathology retrospectively applying two different psychometric measurement scales to compare the psychopathological profile of autoantibody-associated psychiatric syndromes to psychiatric syndromes without autoantibodies. We hypothesize that the psychopathology as measured by the AMDP or HiTOP will differ between autoantibody-positive and autoantibody-negative psychiatric patients. Our second aim is to evaluate the usefulness of the AMDP and HiTOP classifications in terms of their usefulness in detecting specific psychopathologic features that would facilitate differentiating between neural autoantibody-associated psychiatric syndromes from those without autoantibodies. Furthermore, as our third aim, we will correlate the semiquantitative measurement score of antibody intensity with the expression of psychopathology in autoantibody-associated psychiatric syndromes. We hypothesize that the psychopathology as measured by the AMDP or HiTOP will differ between autoantibody-positive and autoantibody-negative psychiatric patients.

## Methods

### Selection of patient cohort

**Figure 1:**
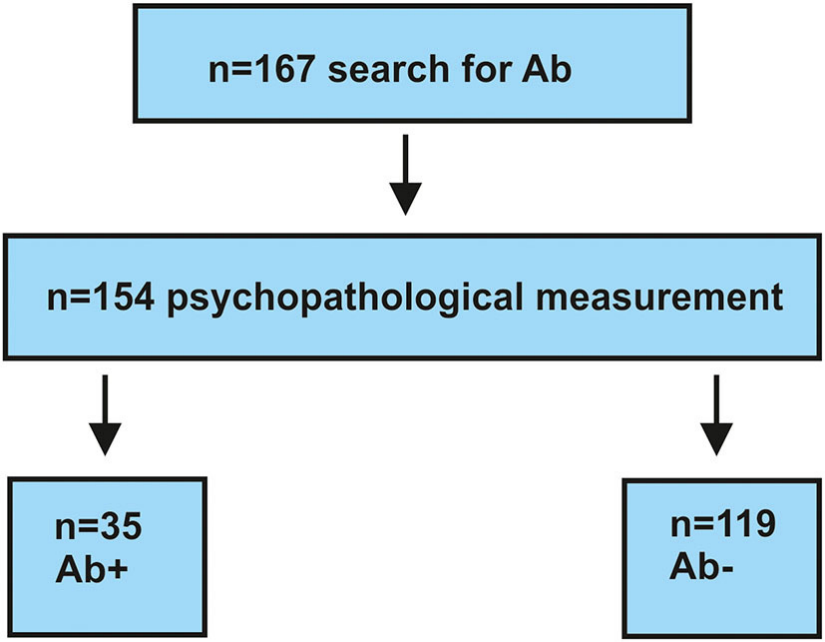
Flow chart for selecting patients. Ab+, autoantibody-positive; Ab–, autoantibody-negative; *n*, number.

We retrospectively screened between 2017 and 2020 a cohort of 167 patients (Figure 1) from the Department of Psychiatry and Psychotherapy, University Medical Center Göttingen. All patients presented different psychiatric diagnoses and were screened for specific autoantibodies (see section autoantibody determination) because we suspected that their psychiatric symptoms had an organic origin. Organic origin has been suspected due to various features associated with the presentation of clinical features with a fulminant course, the acuity of symptoms, the rapid progression of symptoms, the atypical severity of cognitive dysfunction associated with other symptoms or alone, or the presence of autoimmune indicators recently presented in a review (4). Severe cognitive dysfunction including orientation and attention functions, which is not explained otherwise, is a strong indicator than the red flag (5) of an autoimmune base of symptoms and thus another reason for screening neuronal autoantibodies in patients. Our cohort was divided into two subgroups: (1) consisted of 35 serum antibody-positive and (2) contained only 132 serum antibody-negative patients (Figure 1). Our inclusion criterion was a psychiatric disorder for which we had determined autoantibodies for differential diagnostic reasons. We did not exclude patients with comorbidities or additional neurologic conditions. All patients recruited in 2020 had been screened for a CoV-SARS-2 infection. Patients recruited in 2016–2019 did not undergo testing for CoV-SARS-2. Their psychopathology was retrospectively assessed *via* two psychopathological classification systems by studying patient files. Patient data were classified by one rater (IMG) according to the AMPD system (9) and the HiTOP classification system (10, 11). An ultimate total of 154 patients who had undergone complete psychopathological investigation according to AMDP were included in our study. Among the total 154 patients, our groups consisted of 35 serum antibody-positive and 119 serum antibody-negative patients (Figure 1). We used the STrengthening the Reporting of OBservational studies in Epidemiology (STROBE) guidelines to organize and describe our observational study better. Our investigations were approved by our local ethics committee and are in accordance with the Declaration of Helsinki.

### Assessment of psychopathology

Psychopathology was retrospectively assessed from patient files by one rater (IMG). Psychopathological features according to AMDP were assessed as single items from the following domains: disturbances of consciousness, orientation, memory and attention, formal thought disorder, worries and compulsions, delusions, disorders of perception, hallucinations, ego disturbances, disturbances of affect, disorders of drive and psychomotor activity, circadian disturbances, and other disorders. Furthermore, we applied spectra from the HiTOP classification system entailing somatoform, internalizing, thought disorder, disinhibited externalizing, antagonistic externalizing, and detachment spectra. Sexual problems, eating pathologies, fear, distress, mania, substance abuse, and antisocial behavior functioned as HiTOP factors. The two HiTOP superspectra emotional dysfunction (12) and psychosis (13) were also evaluated. The superspectra emotional dysfunction consists of the single spectra somatoform and internalizing, whereas the superspectra psychosis comprises the single spectra detachment and thought disorder. For AMDP domains, we established a total score consisting of the AMDP items from one AMDP domain that were present in the patients. Thus, the total AMDP domain's scoring range is different [disturbances of consciousness (0–4), disturbances of orientation (0–4), disturbances of memory and attention (0–6), formal thought disorder (0–12), worries and compulsions (0–6), delusions (0–6), disorders of perception (0–6), hallucinations (0–6), ego disturbances (0–6), disturbances of affect (0–21), disorders of drive and psychomotor activity (0–9), circadian disturbances, and other disturbances (0–10)]. Moreover, we set up an HiTOP sum score for the superspectra emotional dysfunction and psychosis consisting of the presence of individual spectra present in the patients [psychosis (0–2), emotional dysfunction (0–2)]. Psychopathology was retrospectively assessed from patient files by one rater (IMG).

### Autoantibody determination

Our cerebrospinal fluid samples resulted from lumbar puncture. CSF and blood samples were taken at the same time and were processed according to the standard protocol in the neurochemistry laboratory in the University Medical Center Göttingen's Neurology Department. Freshly extracted cerebrospinal fluid is used, as *in vitro* cytolysis is so rapid. All biomaterial samples were processed within 1–2 h after they were received to ensure accurate cell count samples. The CSF samples were centrifuged to separate cells from CSF at these settings (700 revolutions correspond to 105 × g, 10 min, room temperature). The degeneration marker (ptau181, t-tau) and Reiber scheme were determined on the same day. However, the serum was centrifugated different settings (5,000 revolutions corresponding to 4,528 × g for 5 min at 20°). All serum samples were stored until they were sent as aliquots (500 μl serum) to the Euroimmun laboratory for the detection of autoantibodies. About 300 μl CSF aliquots were also sent to the Euroimmun laboratory. All patients' neural autoantibodies were assessed by the Clinical Immunological Laboratory Prof. Stöcker. We sought diverse neural autoantibodies in peripheral blood (PB) and CSF entailing autoantibodies against membrane cell surface targets [α-amino-3-hydroxy-5-methyl-4-isoxazolepropionic acid receptors 1/2 (AMPAR1/2), Aquaporin 4, contactin-associated protein 2 (CASPR2), dipeptidyl-peptidase-like 6 protein (DPPX), gamma aminobutyric acid B1/2 receptor (GABAB1/2R), glutamic acid decarboxylase (GAD65), leucine-rich glioma inactivated protein 1 (LGI1), and N-methyl-D-aspartate receptor (NMDAR)] and autoantibodies against intracellular often paraneoplastic antigen targets [autoantibodies against intracellular autoantibodies: amphiphysin, CV2, glutamic acid decarboxylase (GAD65), HuD, Ma1/ Ma2, neurochondrin (NC), Ri, TR, Yo, and Zic4]. Euroline immunoblot and cell-based assays were utilized to determine autoantibodies. Neural autoantibodies were assessed in a semiquantitative scale possessing three intensity levels: 1 (mild intensity), 2 (moderate intensity), and 3 (strong intensity). The groups were divided into patients with neural autoantibodies and those without them in serum and or cerebrospinal fluid (CSF). The CSF was analyzed in the Neurochemistry Laboratory, University Medical Center Göttingen.

### Statistical approach

Data were analyzed with SPSS (IBM, SPSS, version 28, Ehningen, Germany). Graphs were made with Sigma Plot (Version 11, Palo Alto, United States). Mean and standard error of the mean were calculated for gender and age. In addition, for the investigation of age differences between men and women, the Mann–Whitney *U*-test was used. The psychopathological items and domains from AMDP as well as factors, superspectra, and spectra from HiTOP classification were compared between autoantibody-positive and autoantibody-negative groups *via* Fisher's exact test with no correction for multiple testing because of the data exploratory nature and our small sample. We conducted no power analysis beforehand, as our retrospective cohort analysis has exploratory character. We used Spearman's rho correlation including correcting for multiple testing *via* Bonferroni to investigate the relationship between the semiquantitative autoantibody-intensity score and psychopathological items sum score of the AMDP system and HiTOP classification. To estimate effect sizes and our results' robustness, we also conducted Bayesian testing and calculated the effect sizes *via* Cramer's V. A *p*-level of < 0.05 was considered as significant.

## Results

### Demographics and clinical characteristics of patients

We enrolled 154 patients in this study with the following diagnostic spectrum shown in Table 1. In 35 of 154 (22.7%) psychiatric patients, we detected a neural antibody in the peripheral blood (Ab+ PB), and 13 of 154 (8.5%) had antibodies detected in CSF (Ab+ CSF). We divided the patients in a group of 35 psychiatric patients with peripheral blood autoantibodies (PsychAb + PB) and 119 psychiatric patients with no PB autoantibodies (PsychAb – PB). A patient classification found that 27 out of 35 (78%) of serum autoantibody-positive patients had possible autoimmune encephalitis and six out of 35 (17%) had definitive autoimmune encephalitis according to the criteria of Graus et al. (1), while seven out of 35 (20%) showed autoimmune psychosis according to the criteria of Pollak et al. (14) (Table 2). A lumbar puncture could not be performed in four patients with serum autoantibody detection and in three patients without serum autoantibody detection. Our patient groups consisted of various psychiatric diagnoses whose main diagnoses did not differ in their frequency between groups (Table 1). PsychAb + PB patients were diagnosed with disorders belonging to organic, including symptomatic, mental disorders (60%, *n* = 24), schizophrenia, schizotypical and delusional disorders (3%, *n* = 1), mood disorders (5%, *n* = 14), anxiety, dissociative, stress-related, somatoform, and other nonpsychotic mental disorders (4%, *n* = 2). On the contrary, PsychAb – PB patients were diagnosed with organic, including symptomatic, mental disorders (45%, *n* = 53), schizophrenia, schizotypical and delusional disorders (18%, *n* = 15), mood disorders (35%, *n* = 29), anxiety, dissociative, stress-related, somatoform and other nonpsychotic mental disorders (4%, *n* = 2), and adult personality and behavior disorders (2%, *n* = 2) (Table 1). The psychiatric diagnoses were made after the differential diagnostic approach was carried out, including the determination of neuronal autoantibodies. We formed a group of 13 of 154 (8.5%) patients who presented CSF neural autoantibodies (PsychAb + CSF) (Table 2) and a corresponding group of 141 patients with no CSF autoantibodies (PsychAb – CSF). See for autoantibodies of the PsychAb + PB group, serum and CSF group as well as CSF-only group autoantibodies (Table 2). In total, our cohort consisted of 80 women and 74 men. The men had a mean age of 54 years (54.3 ± 2.1 years), and the women were 61 years old (61.2 ± 1.7 years). However, the age of men and women did not differ throughout our entire cohort, the PsychAb + PB and PsychAb – PB patients. Age and gender did not differ between groups (PsychAb + PB vs. PsychAb – PB, and PsychAb + CSF vs. PsychAb – CSF) (Table 3). Several psychiatric syndromes did not differ among the (PsychAb + PB vs. PsychAb – PB, and PsychAb + CSF vs. PsychAb – CSF) groups (Table 3). However, many more psychiatric patients with a manic syndrome according to the AMDP classification presented neural autoantibodies (PsychAb+ or PsychAb + CSF) (8.6%) compared to neural autoantibody-negative psychiatric patients (PsychAb – PB or PsychAb – CSF) (0.8%) (*p* < 0.05) (Table 3).

**Table 1:** Composition of psychiatric diagnosis in cohort of patients.

**Category of psychiatric disorder ICD10**	**Complete cohort**	**Complete cohort**	**Ab+**	**Ab+**	**Ab–**	**Ab–**
			***n***	**%**	***n***	**%**
Organic, including symptomatic, mental disorders, F00–F09	77/154	50	24/35	69	53/119	45
Mental and behavioral disorders due to psychoactive substance use, F10–F19	4/154	3	–	–	4/119	3
Schizophrenia, schizotypical and delusional disorders, F20–F29	19/154	12	1/35	3	18/119	15
Mood disorders, F30–F39	40/154	26	5/35	14	35/119	29
Anxiety, dissociative, stress-related, somatoform and other nonpsychotic mental disorders, F40–F49	6/154	4	2/35	4	4/119	3
Adult personality and behavior disorders, F60–F69	2/154	1	–	–	2/119	2

Ab–, antibody negative; Ab+, antibody positive; ICD10, International Classification of Diseases, Tenth Revision; *N*, number.

**Table 2:** Classification of neural autoantibody-positive patients.

**Patient number**	**Ab serum**	**Ab CSF**	**Possible AE**	**Definitive AE**	**Autoimmune psychosis**
1	Ma2	Ma2	Present	Not-present	Present
2	CV2/CRMP5	CV2/CRMP5	Present	Present	Present
3	Yo	Not present	Not present	Not present	Not present
4	Myelin	Not present	Present	Not present	Not present
5	NMDAR	Not present	Present	Not present	Not present
6	NMDAR		Present	Present	Present
7	NC and Titin	NC and Titin	Not present	Not present	Not present
8	Not present	Yo	Present	Present	Not present
9	Neuropil (unspecific)	Not present	Present	Not present	Not present
10	GlycineR	Not present	Present	Not present	Not present
11	GAD65, Recoverin	Not present	Present	Not present	Not present
12	GAD65, TR/DNER, Yo	–	Present	Not present	Not present
13	Recoverin	Not present	Not present	Not present	Not present
14	KCNA2	KCNA2	Present	Not present	Not present
15	Myelin	Not present	Present	Not present	Not present
16	Titin, Yo	Not present	Present	Present	Not present
17	Recoverin	–	Present	Not present	Not present
18	NMDAR	NMDAR	Present	Present	Not present
19	Zic4	–	Not present	Not present	Not present
20	NMDAR	–	Not present	Not present	Not present
21	Yo	Not present	Present	Not present	Not present
22	GlycineR	Not present	Not present	Not present	Not present
23	Amphiphysin	Not present	Not present	Not present	Not present
24	KCNA2	Not present	Not present	Not present	Not present
25	CV2	Not present	Present	Not present	Not present
26	NMDAR	Not present	Present	Present	Present
27	NMDAR	NMDAR	Present	Present	Present
28	CASPR2	CASPR2	Present	Present	Present
29	Zic4, SOX, Ma1	Zic4, SOX, Ma1	Present	Not present	Present
30	NMDAR	NMDAR	Present	Not present	Not present
31	Myelin	Not present	Present	Not present	Not present
32	Titin	Titin	Present	Not present	Not present
33	Neuropil (unspecific)	Neuropil (unspecific)	Present	Not present	Not present
34	Recoverin	–	Present	Not present	Not present
35	CASPR2	Not present	Not present	Not present	Not present
36	IgLON5	IgLON5	Present	Present	Not present

AE, autoimmune encephalitis; CASPR2, contactin-associated protein-2; CV2/CRMP5, cronveinten 2/Collapsin response mediator protein 5; GAD65, glutamic acid decarboxylase of kDa65; KCNA2, Potassium Voltage-Gated Channel Subfamily A Member 2; NMDAR, N-methyl-D-aspartate receptor; NC, Neurochondrin; SOX1, Recoverin, sry-like high motility group box 1; Zic4, zinc finger protein of the cerebellum 4; Ab PB, autoantibody peripheral blood; Ab CSF, autoantibody cerebrospinal fluid; –, no CSF analysis done.

**Table 3:** Demographics and clinical data of patient groups.

	**PsychAb** + **PB**		**PsychAb** – **PB**		***p*-value**	**PsychAb** + **CSF**		**PsychAb** – **CSF**		***p*-value**
***n***	**35**		**119**			**13**		**141**		
**Gender**	**M**	**F**	**M**	**F**		**M**	**F**	**M**	**F**	
*n* (%)	19/35 (54%)	16/35 (46%)	55/119 (46%)	64/119 (54%)		8/13 (62%)	5/13 (38%)	66/141 (47%)	75/141 (53%)	
Age mean value (range)	61.3 (55.9–66.7)		56.9 (53.7–60.1)		0.241	59.5 (48.8–70.3)		57.72 (54.9–60.6)		0.701
**Psychiatric syndrome**	
Paranoid hallucinatory, *n* (%)	9/35 (25.70%)		37/119 (31.10%)		0.349	7/13 (53.80%)		39/141 (27.70%)		0.053
Depressive, *n* (%)	21/35 (60.0)		74/119 (62.2%)		0.482	6/13 (46.2%)		89/141 (63.1%)		0.182
Manic, *n* (%)	3/35 (8.6%)		1/119 (0.8%)		<0.05	2/13 (15.4%)		2/141 (1.4%)		<0.05
Apathic, *n* (%)	4/35 (11.4%)		15/119 (12.6%)		0.558	2/13 (15.4%)		17/141 (2.1%)		0.497
Vegetative, *n* (%)	1/35 (2.90%)		8/119 (6.70%)		0.351	1/13 (7.70%)		8/141 (5.7%)		0.558
Hostility	4/35 (1.40%)		7/119 (5.90%)		0.22	2/13 (15.40%)		9/141 (6.4%)		0.234
Neurological, *n* (%)	4/35 (11.4%)		17/119 (14.3%)		0.454	1/13 (7.7%)		20/141 (14.2%)		0.445
Psychorganic, *n* (%)	30/35 (85.7%)		88/119 (73.9%)		0.109	11/13 (84.6%)		107/141 (75.9%)		0.375

*N*, number of patients; PsychAb + CSF, psychiatric patients with cerebrospinal fluid neural autoantibodies; PsychAb + PB, psychiatric patients with serum neural autoantibodies; PsychAb – CSF, psychiatric patients without cerebrospinal fluid neural autoantibodies; PsychAb – PB, psychiatric patients without serum neural autoantibodies; M, male; F, female.

### Psychiatric patients with neural serum autoantibodies

#### AMDP classification

**Figure 2:**
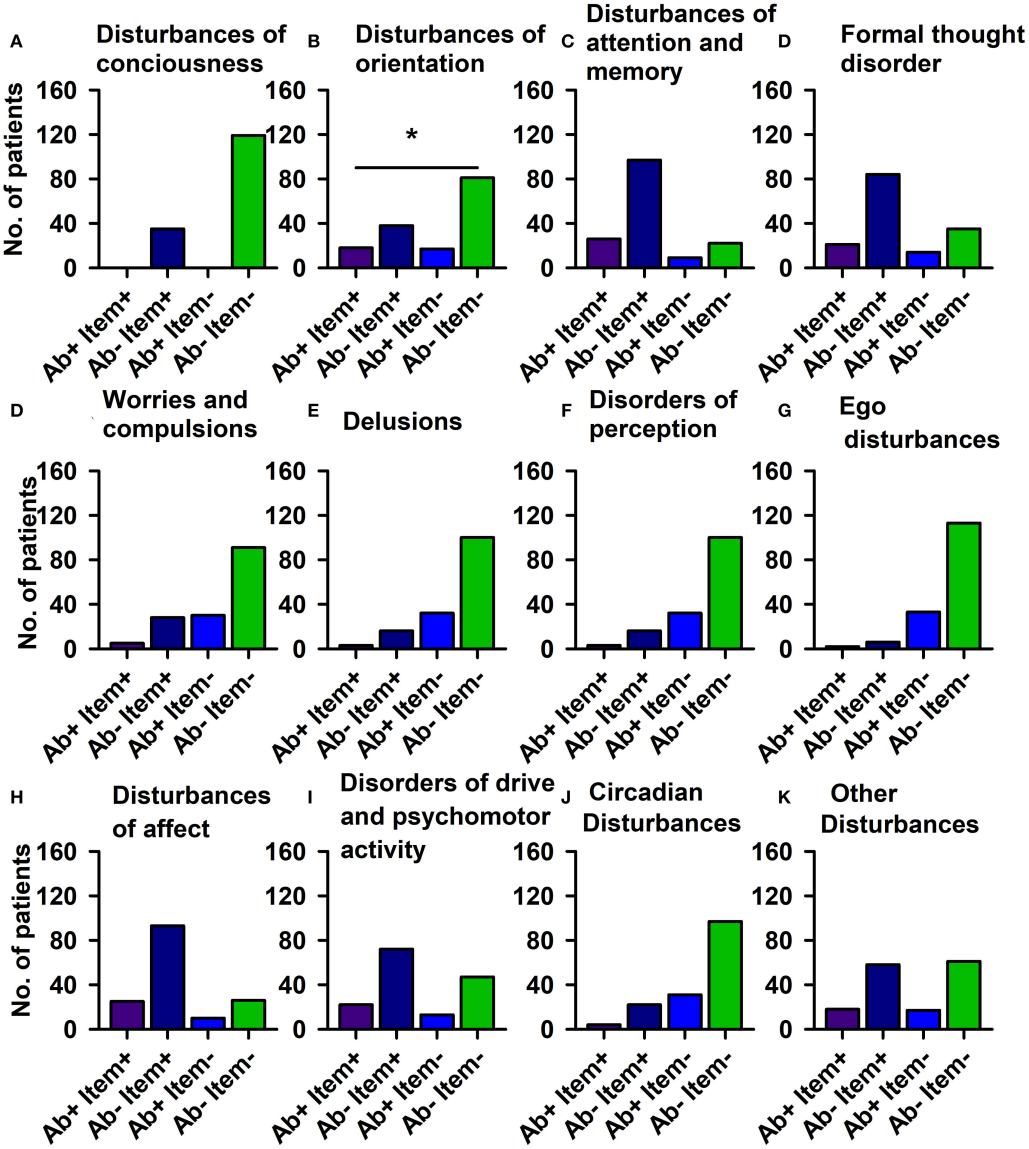
Psychopathology domains of psychiatric patients with serum neural autoantibodies vs. those without — AMDP system. Significant orientation disturbances are observed in serum neural autoantibody-positive psychiatric patients compared to those with no autoantibodies **(B)**. The other domains **(A,C–K)** reveal no relevant differences between groups. Ab+, psychiatric patients with neural autoantibodies; Ab–, psychiatric patients without neural autoantibodies; AMDP, Arbeitsgemeinschaft für Methodik und Dokumentation in der Psychiatrie; item+, item is present; item–, item absent; No., number. *p < 0.05 Fisher's exact test.

**Figure 3:**
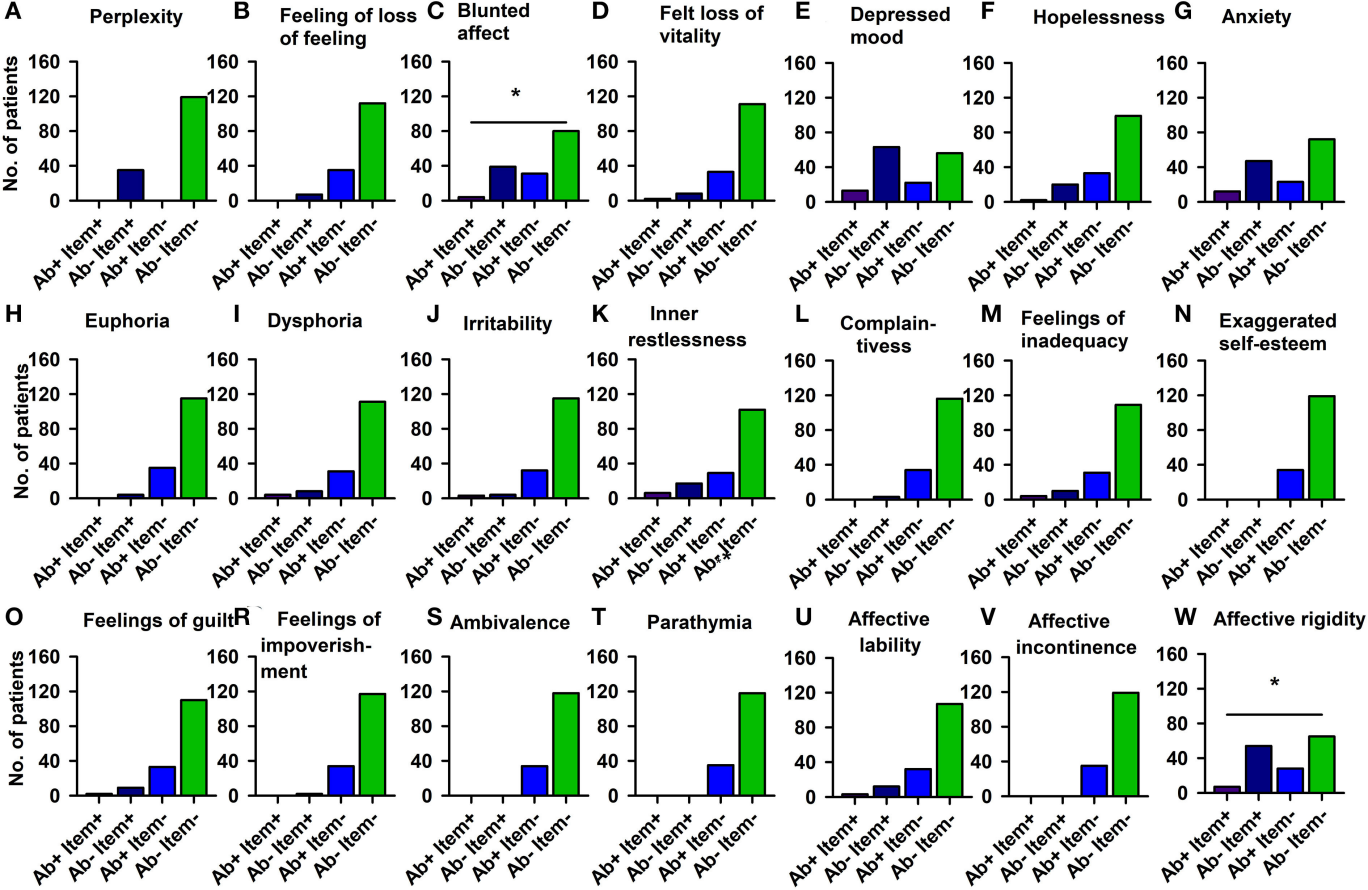
Affective disturbances of psychiatric patients with serum neural autoantibodies vs. those without them — AMDP system. Significantly less blunted affect **(C)** and less affective rigidity **(W)** appears in serum neural autoantibody-positive psychiatric patients compared to those with no autoantibodies. The other domains **(A,B,D–V)** show no relevant differences between groups. Ab+, psychiatric patients with neural autoantibodies; Ab–, psychiatric patients without neural autoantibodies; AMDP, Arbeitsgemeinschaft für Methodik und Dokumentation in der Psychiatrie; item+, item is present; item–, item absent; No., number. *p < 0.05 Fisher's exact test.

Using domains of the AMDP system, our analysis revealed more orientation disorders (51.4%) in our PsychA b +PB than the PsychAb – PB group (31.9%) (Figure 2; Table 4A, *p* < 0.05, Cramer's V: 0.170, Cronbach's alpha: 0.807, Bayesian test: 2.7, log odds ratio: 0.81). However, other AMDP criteria like disturbances of consciousness, attention and memory disorder, formal thought disorder, worries and compulsions, delusions, hallucinations, ego disturbances, affective disorders, drive and psychomotor disorders, circadian, and other disorders did not differ between PsychAb + PB and PsychAb – PB patients (Figure 2; Table 4A). When looking closer at the AMDP system's affective characteristics, PsychAb + PB (11.4%) showed less blunted affect than PsychAb – PB patients (32.8%) (*p* < 0.01, Cramer's V: 0.199, Bayesian test: 7.3, log odds ratio: −1.23, Figure 3; Table 4B). Furthermore, the affective rigidity was more pronounced in PsychAb– (45.4%) than PsychAB + patients (20%, *p* < 0.01, Cramer's V: 0.22, Bayesian test: 13.44, log odds ratio: −1.14; Figure 3; Table 4B). However, no other affective symptoms were revealed such as the feeling of perplexity, loss of feeling, feeling loss of vitality, depressive mood, hopelessness, anxiety, euphoria, dysphoria, irritability, inner restlessness, complaining, feelings of insufficiency, increased self-esteem, feelings of guilt, feelings of impoverishment, ambivalence, parathymia, affect lability, affective incontinence, and lack of effects (Figure 3; Table 4B).

**Table 4:** (A–D) Psychopathology of psychiatric patients presenting serum or cerebrospinal fluid neural autoantibodies versus those without them – AMDP domains and affective symptoms.

**A**	**PsychAb –PB**		**PsychAb**+**AB**		**Fischer's exact test**	**Effect size**	**Cronbach's**	**Bayesian test**	**Log odds ratio**
**AMDP domains**	**Not present**	**Present**	**Not present**	**Present**	***p-value***	**Cramers V**	**Alpha**	**BF 10 poisson**	
Disturbance of consciousness	100%	–	100%	–		–	–	–	–
Disturbances of orientation	68.1%	31.9%	48.6%	51.4%	<0.05	0.170	0.807	2.780	0.806 (0.059/1.554)
Disturbances of memory and attention	18.5%	81.5%	25.7%	74.4%	0.239	0.076	0.583	0.428	−0.437 (−1.311/0.437)
Formal thought disorders	29.4%	70.6%	40%	60%	0.164	0.095	0.307	0.626	−0.477 (−1.247/0.293)
Worries and compulsions	76.5%	23.5%	85.7%	14.4%	0.174	0.094	0.210	0.504	−0.551 (−1.564/0.463)
Delusions	84%	16%	91.4%	8.6%	0.209	0.089	0.804	0.385	−0.595 (−1.797/0.607)
Disorders of perception	84%	16%	91.4%	8.6%	0.209	0.089	0.417	0.385	−0.586 (−1.781/0.610)
Ego disturbances	95%	5%	94.3%	5.7%	0.579	0.013	0.431	0.159	0.281 (−1.214/−1.776)
Disturbances of affect	21.8%	78.2%	28.6%	71.4%	0.27	0.067	0.636	0.407	−0.365 (−1.201/0.471)
Disorders of drive and psychomotor activity	39.5%	60.5%	37.1%	62.9%	0.482	0.020	0.054	0.331	0.091 (−0.768/0.859)
Circadian disturbances	81.5%	18.5%	88.6%	11.4%	0.24	0.079	−	0.370	−0.483 (−1.568/0.601)
Other disturbances	51.3%	48.7%	48.6%	51.4%	0.465	0.023	0.309	0.343	0.111 (−0.635/0.856)
**B**	**PsychAb –PB**		**PsychAb**+**AB**		**Fischer's exact test**	**Effect size**	**Bayesian test**	**Log odds ratio**
**AMDP items affectivity disturbances**	**Not present**	**Present**	**Not present**	**Present**	***p-value***	**Cramers V**	**Alpha**	**BF 10 poisson**	
Perplexity	94.1%	5.9%	97.1%	2.9%	0.421	0.057	0.167	−0.419 (−2.195 /1.357)
Feeling of loss of feeling	94.1%	5.9%	100%	–	0.158	0.118	0.319	−1.432 (−4.077/1.212)
Blunted affect	67.2%	32.8%	88.6%	11.4%	<0.01	0.199	7.299	−1.232 (−2.265/-0.198)
Feeling loss of vitality	39.3%	6.7%	94.3%	5.7%	0.594	0.017	0.167	−0.419 (−2.195/1.357)
Depressed mood	47.1%	52.9%	62.9%	37.1%	0.073	0.132	1.247	−0.626 (−1.393/0.140)
Hopelessness	83.2%	16.8%	94.3%	5.7%	0.078	0.133	0.847	−1.026 (−2.408/0.356)
Anxiety	60.5%	39.5%	65.7%	34.3%	0.363	0.045	0.369	−0.210 (−0.985/0.566)
Euphoria	96.6%	3.4%	100%	–	0.352	0.089	0.143	−0.898 (−3.604−1.807)
Dysphoria	93.3%	6.7%	88.6%	11.4%	0.277	0.074	0.293	0.636 (−0.566/1.837)
Irritability	96.6%	3.4%	91.4%	8.6%	0.194	0.105	0.334	1.018 (−0.425/2.460)
Inner restlessness	85.7%	14.3%	82.9%	17.1%	0.428	0.034	0.268	0.261 (−0.728/1.249)
Complaintiveness	97.5%	2.5%	97.1%	2.9%	0.648	0.009	0.118	0.400 (−1.508/2.307)
Feelings of inadequacy	91.6%	8.4%	88.6%	11.4%	0.397	0.044	0.238	0.399 (−0.754/1.553)
Exaggerated self-esteem	100%	−	97.1%	2.9%	0.227	0.149	0.221	2.222 (−0.754/5.198)
Feelings of guilt	92.4%	7.6%	94.3%	5.7%	0.524	0.030	0.176	−0.137 (−1.593/1.318)
Feelings of impoverishment	98.3%	1.7%	97.1%	2.9%	0.541	0.036	0.122	0.714 (−1.336/2.763)
Ambivalence	99.2%	0.8%	97.1%	2.9%	0.404	0.075	0.142	1.234 (−1.011/3.479)
Parathymia	99.2%	0.8%	100%	−	0.773	0.044	0.065	0.220 (−2.760- 3.199)
Affective lability	89.9%	10.1%	91.4%	8.6%	0.543	0.021	0.199	−0.076 (−1.325/1.173)
Affective incontinence	100%	−	100%	−		−	−	−
Affective rigidity	54.6%	45.4%	80%	20%	<0.01	0.217	13.446	−1.144 (−2.024/−0.264)
**C**	**PsychAb –CSF**		**PsychAb**+**CSF**		**Fischer's exact test**	**Effect size**	**Bayesian test**	**Log odds ratio**
**AMDP criterion**	**Not present**	**Present**	**Not present**	**Present**	***p-value***	**Cramers V**	**Alpha**	**BF 10 poisson**	
Disturbance of consciousness	100%	–	100%	–	–	–		
Disturbances of orientation	65.2%	34.8%	46.2%	53.8%	0.143	0.110	0.539	0.779 (−0.332/1.890)
Disturbances of memory and attention	19.9%	80.1%	23.1%	76.9%	0.508	0.022	0.195	−0.287 (−1.561/0.988)
Formal thought disorders	32.6%	67.4%	23.1%	76.9%	0.357	0.057	0.247	0.381 (−0.888/1.649)
Worries and compulsions	79.4%	20.6%	69.2%	30.8%	0.293	0.069	0.284	0.587 (−0.615/1.788)
Delusions	86.5%	13.5%	76.9%	23.1%	0.277	0.076	0.274	0.738 (0.572/2.047)
Disorders of perception	85.1%	14.9%	92.3%	7.7%	0.417	0.057	0.171	−0.357 (−2.061/1.347)
Ego disturbances	95%	5%	92.3%	7.7%	0.515	0.034	0.134	0.744 (−1.043/2.591)
Disturbances of affect	23.4%	76.6%	23.1%	76.9%	0.641	0.002	0.189	−0.067 (−1.330/1.196)
Disorders of drive and psychomotor activity	40.4%	59.6%	23.1%	76.9%	0.177	0.099	0.425	0.706 (−0.459/1.960)
Circadian disturbances	83.7%	16.3%	76.9%	23.1%	0.380	0.050	0.223	0.525 (−0.770/1.820)
Other disturbances	51.8%	48.2%	38.5%	61.5%	0.265	0.074	0.330	0.510 (−0.614/1.635)
**D**	**PsychAb –CSF**		**PsychAb**+**CSF**		**Fischer's exact test**	**Effect size**	**Bayesian test**	**Log odds ratio**
**AMDP criterion affectivity disorders**	**Not present**	**Present**	**Not present**	**Present**	***p-value***	**Cramers V**	**Alpha**	**BF 10 poisson**	
Perplexity	95%	5%	92.3%	7.7%	0.515	0.034	0.134	0.777 (−0.031/2.586)
Feeling of loss of feeling	95%	5%	100%	–	0.532	0.066	0.094	−0.280 (−2.953/2.392)
Blunted affect	70.2%	29.8%	92.3%	7.7%	0.076	0.137	0.776	−1.254 (−2.958/0.450)
Fell loss of vitality	95%	5%	76.9%	23.1%	<0.05	0.204	1.428	1.792 (0.396/3.188)
Depressed mood	48.9%	51.1%	69.2%	30.8%	0.133	0.113	0.562	−0.781 (−1.967/0.394)
Hopelessness	85.1%	14.9%	92.3%	7.7%	0.417	0.057	0.171	−0.363 (−2.103/1.378)
Anxiety	61.7%	38.3%	61.5%	38.5%	0.605	0.001	0.216	0.039 (−1.082/1.159)
Euphoria	97.2%	2.8%	100%	–	0.700	0.050	0.072	0.237 (−2.457/2.930)
Dysphoria	92.2%	7.8%	92.3%	7.7%	0.732	0.001	0.126	0.323 (−1.443/2.089)
Irritability	96.5%	3.5%	84.6%	15.4%	0.109	0.158	0.530	1.675 (0.045/3.306)
Inner restlessness	85.8%	14.2%	76.9%	23.1%	0.303	0.069	0.257	0.672 (−0.632/1.975)
Complaintiveness	97.9%	2.1%	92.3%	7.7%	0.300	0.097	0.191	1.579 (−0.385/3.543)
Feelings of inadequacy	90.1%	9.9%	100%	–	0.275	0.096	0.183	−1.016 (−3.672/1.639)
Exaggerated self-esteem	100%	–	92.3%	7.7%	0.084	0.266	0.596	3.408 (0.407/6.408)
Feelings of guilt	92.2%	7.8%	100%	–	0.366	0.084	0.137	−0.754 (−3.428/1.921)
Feelings of impoverishment	97.9%	2.1%	100%	–	0.766	0.043	0.066	0.500 (−2.255/3.256)
Ambivalence	98.6%	1.4%	100%	–	0.838	0.035	0.060	0.852 (−1.964/3.667)
Parathymia	99.3%	0.7%	100%	–	0.916	0.025	0.055	1.327 (−1.722/4.377)
Affective lability	90.1%	9.9%	92.3%	7.7%	0.631	0.021	0.132	0.092 (−1.634/1.819)
Affective incontinence	100%	–	100%	–		–	–	-
Affective rigidity	58.9%	41.1%	76.9%	23.6%	0.165	0.103	0.452	−0.736 (−1.992/0.519)

AMDP, Arbeitsgemeinschaft für Methodik und Dokumentation in der Psychiatrie; PsychAb-PB, psychiatric patients without peripheral blood autoantibodies; PsychAb+AB, psychiatric patients with peripheral blood autoantibodies.

#### HiTOP classification

We found no differences between PsychAb + PB and PsychAb – PB groups in various spectra (somatoform, internalizing, thought disorder, disinhibited externalizing, and antagonistic externalizing) from the HiTOP classification (Table 5A; Supplementary Figure 1). Furthermore, HiTOP classification's frequency subfactors did not distinguish PsychAb + PB from PsychAb – PB patients (Table 5B; Supplementary Figure 2).

**Table 5:** (A–F) Psychopathology of psychiatric patients presenting serum or cerebrospinal fluid neural autoantibodies versus those without them – HiTOP spectra, factors and superspectra.

**A**	**PsychAb –PB**		**PsychAb** +**PB**		**Fischer's exact test**	**Effect size**	**Cronbachs**	**Bayesian test**	**Log odds ratio**
**HiTOP spectra**	**No present**	**Present**	**No present**	**Present**	***p-value***	**Cramers V**	**Alpha**	**BF 10 poisson**	
Somatoform	84.9%	15.1%	94.3%	5.7%	0.117	0.117	0.252	0.583	−0.883 (−2.247−0.482)
Internalizing	52.9%	47.1%	68.6%	31.7%	0.073	0.132	−	1.226	−0.644 (−1.433−0.145)
Thought disorder	79%	21%	82.9%	17.1%	0.407	0.040	0.163	0.209	−0.207 (−1.169−0.754)
Disinhibited externalizing	88.2%	11.8%	91.4%	8.6%	0.429	0.043	−	0.225	−0.231 (−1.451−0.990)
Antagonistic externalizing	96.6%	3.4%	100%	−	0.352	0.089	0.025	0.143	−0.898 (−3.572−1.777)
Detachment	92.4%	7.6%	100%	−	0.091	0.135	0.184	0.550	−1.673 (−4.306−0.959)
**B**	**PsychAb –PB**		**PsychAb** +**PB**		**Fischer's exact test**	**Effect size**	**Cronbachs**	**Bayesian test**	**Log odds ratio**
**HiTOP factors**	**No present**	**Presnt**	**No present**	**Present**	***p-value***	**Cramers V**	**Alpha**	**BF 10 poisson**	
Sexual problems	96.9%	3.4%	100%	−	0.352	0.089	−	0.143	−0.912 (−3.633/1.809)
Eating pathology	100%	0%	100%	−	−	−	−	−	-
Fear	89.1%	10.3%	97.1%	2.9%	0.126	0.118	0.113	0.485	−1.051 (−2.739/0.636)
Distress	60.5%	39.5%	74.3%	25.7%	0.097	0.120	−	0.933	−0.599 (−1.423/0.224)
Mania	96.6%	3.4%	94.3%	5.7%	0.413	0.051	−	0.179	0.658 (−0.928/2.244)
Substance abuse	89.1%	10.9%	94.3%	5.7%	0.290	0.074	−	0.266	−0.525 (−1.916/0.865)
Antisocial behavior	99.2%	0.8%	97.1%	2.9%	0.404	0.075	0.025	0.142	1.239 (−1.028/3.507)
**C**	**PsychAb –CSF**		**PsychAb** +**CSF**		**Fischer's exact test**	**Effect size**	**Bayesian test**	**Log odds ratio**
**HiTOP spectra**	**Not present**	**Present**	**Not present**	**Present**	***p-value***	**Cramers V**	**BF 10 poisson**	
Somatoform	87.2%	12.8%	84.6%	15.4%	0.526	0.022	0.168	0.375 (−1.067/1.817)
Internalizing	56%	44%	61.5%	38.5	0.468	0.031	0.233	−0.196 (−1.322/0.930)
Thought disorder	80.9%	19.1%	69.2	30.8	0.250	0.081	0.318	0.679 (−0.506/1.865)
Disinhibited externalizing	88.7%	11.3%	92.3%	7.7%	0.566	0.032	0.139	−0.072 (−1.824/1.680)
Antagonistic externalizing	97.2%	2.8%	100%	−	0.700	0.050	0.072	0.236 (−2.525/2.997)
Detachment	93.6%	6.4%	100%	−	0.442	0.076	0.114	−0.544 (−3.201/2.114)
**D**	**PsychAb –CSF**		**PsychAb** +**CSF**		**Fischer's exact test**	**Effect size**	**Bayesian test**	**Log odds ratio**
**HiTOP factors**	**Not present**	**Present**	**Not present**	**Present**	***p-value***	**Cramers V**	**BF 10 poisson**	
Sexual problems	97.9%	2.1%	92.3%	7.7%	0.300	0.097	0.191	1.677 (−0.393/3.548)
Eating pathology	100%	−	100%	−	−	−	−	-
Fear	90.8%	9.2%	92.3%	7.7%	0.665	0.015	0.129	0.129 (−1.650/1.907)
Distress	63.1%	36.9%	69.2%	30.8%	0.455	0.035	0.228	−0.229 (−1.423/0.965)
Mania	96.5%	3.5%	92.3%	7.7%	0.416	0.060	0.150	1.132 (−0.754/3.018)
Substance abuse	89.4%	10.6%	100%	−	0.249	0.100	0.202	−1.085 (−3.78/1.589)
Antisocial behavior	99.3%	0.7%	92.3%	7.7%	0.162	0.171	0.323	2.437 (0.160/4.714)
**E**	**PsychAb –PB**		**PsychAb** +**PB**		**Fischer's exact test**	**Effect size**	**Bayesian test**	**Log odds ratio**
**HiTOP superspectra**	**Not present**	**Present**	**Not present**	**Present**	***p-value***	**Cramers V**	**BF 10 poisson**	
Psychosis	76.5%	23.5%	80%	20%	0.426	0.035	0.297	−0.170 (−1.098/0.758)
Emotional dysfunction	52.1%	47.9%	68.6%%	31.4%	0.062	0.139	1.466	−0.677 (−1.466/0.113)
**F**	**PsychAb –CSF**		**PsychAb** +**CSF**		**Fischer's exact test**	**Effect size**	**Bayesian test**	**Log odds ratio**
**HITOP superspectra**	**Not present**	**Present**	**Not present**	**Present**	***p-value***	**Cramers V**	**BF 10 poisson**	
Psychosis	78.7%	21.3%	61.5%	38.5%	0.143	0.114	0.517	0.864 (−0.282/2.010)
Emotional dysfunction	55.3%	44.7%	61.5%	38.5%	0.448	0.035	0.238	−0.224 (−1.346/0.898)

HiTOP, Hierarchical Taxonomy of Psychopathology; PsychAb+AB, psychiatric patients with peripheral blood autoantibodies; PsychAb –PB, psychiatric patients without peripheral blood autoantibodies; PsychAb+AB, psychiatric patients with peripheral blood autoantibodies; PsychAb-CSF, psychiatric patients without cerebrospinal fluid autoantibodies; PsychAb+CSF, psychiatric patients with cerebrospinal fluid autoantibodies.

### Psychiatric patients with cerebrospinal fluid neural autoantibodies

#### AMDP classification

**Figure 4:**
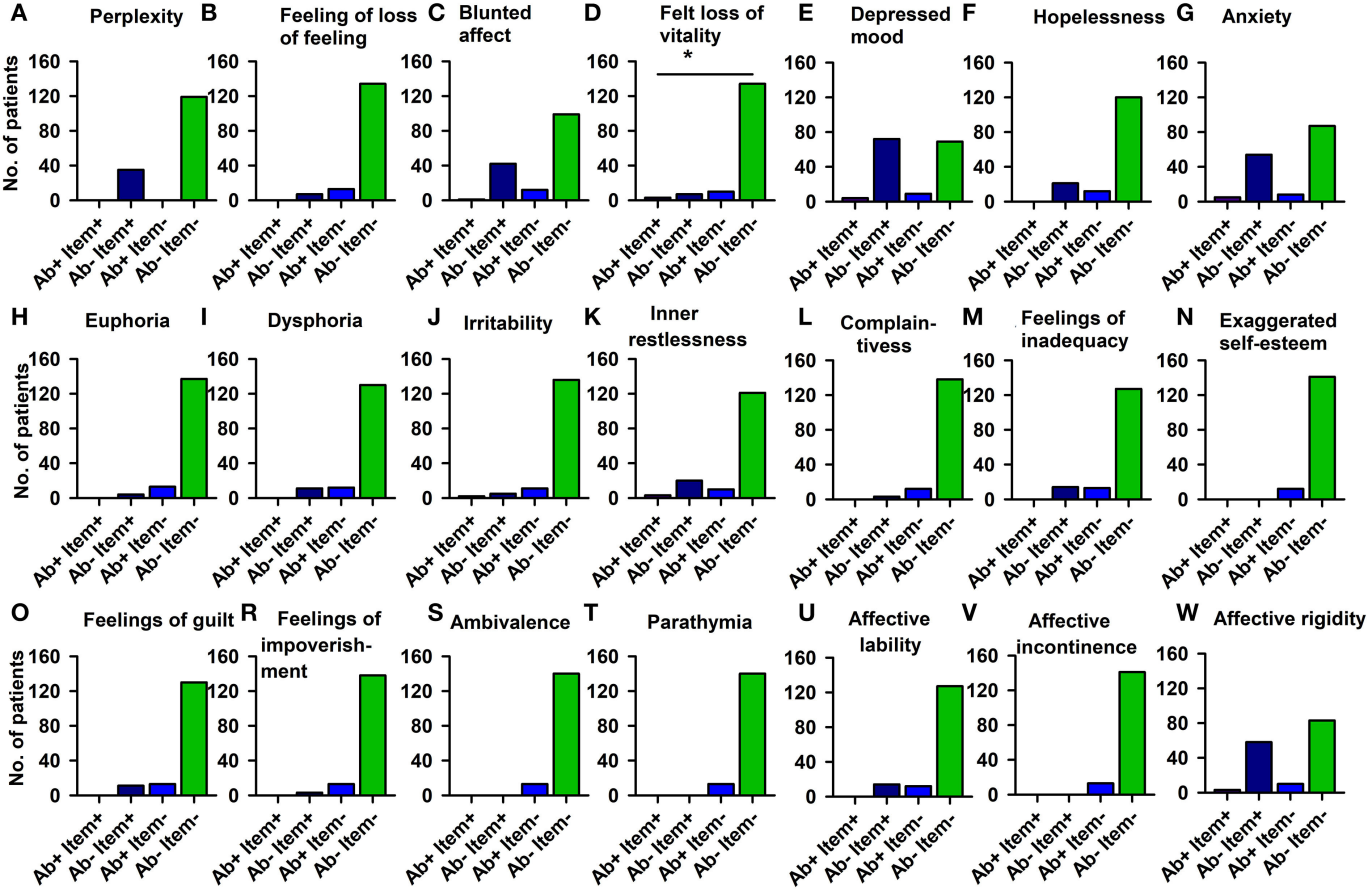
Affective disturbances of psychiatric patients presenting CSF neural autoantibodies vs. those without them — AMDP system. Significantly less feeling of a loss of vitality is depicted in CSF neural autoantibody-positive psychiatric patients compared to those with no autoantibodies **(D)**. The other domains **(A–C,E–W)** show no relevant differences between groups. Ab+, psychiatric patients with neural autoantibodies; Ab–, psychiatric patients without neural autoantibodies; AMDP, Arbeitsgemeinschaft für Methodik und Dokumentation in der Psychiatrie; item+, item is present; item–, item absent; No., number. *p < 0.05 Fisher's exact test.

The PsychAb + CSF patients could not be distinguished from PsychAb – CSF patients in their AMDP psychopathology domains such as orientation disorder, disturbances of consciousness, attention and memory disorder, formal thinking disorder, apprehensions and compulsions, delusions, hallucinations, ego disturbances, affective disorders, drive and psychomotor disorders, and circadian and other disorders (Table 4C). But if affective symptoms are inspected more carefully, the feeling of lost vitality was much stronger in PsychAb + CSF (5%) than PsychAb – CSF patients (0%) (*p* < 0.05, Cramer's V: 0,204, Figure 4; Table 4D).

#### HiTOP classification

The differences in specific psychopathologic symptoms revealed by analyzing the HiTOP classification were not apparent if HiTOP spectra between PsychAb + CSF vs. PsychAb – CSF patients (somatoform, internalizing, thought disorder, disinhibited externalizing, antagonistic externalizing, and detachment) were analyzed *(*Table 5C; Supplementary Figure 3). In addition, the frequency of HiTOP classification factors did not distinguish PsychAb + PB from PsychAb – PB patients (Table 5D; Supplementary Figure 5). We ran an additional analysis to seek superspectra differences between patient groups. The superspectra emotional dysfunction and psychosis did not differ between PsychAb + PB and PsychAb – PB patients, or between PsychAb+ and PsychAb – CSF patients (Tables 5E,F).

### Correlation between serum and CSF neural autoantibody intensity and psychopathological features

We did not find relevant correlations between neural autoantibody intensity in serum (*n* = 27), neural autoantibody intensity in CSF (*n* = 5), combined neural autoantibody intensity in serum and CSF (*n* = 9), and the respective AMDP domain sum score, HiTOP spectrum score, HiTOP subfactor score, and HiTOP superspectrum scores.

### Autoantibody subgroup analysis

To look for differences between autoantibody subtypes, we compared psychiatric patients with autoantibodies against membrane surface targets (*n* = 15) with autoantibodies against intracellular antigens (*n* = 17). We deleted these patients with the detection of not one, but various neural autoantibodies (*n* = 3). Surprisingly, we found more attention and memory impairment in patients with intracellular autoantibodies than in patients with antibodies to membrane surface antigens (Table 6A, Fisher's exact test: *p* < 0.005, Bayesian BF Poisson = 55.30). In affective psychopathology assessed by AMDP, it was surprisingly noticeable that patients with membrane surface autoantibodies did not have blunted affect in contrast to patients with autoantibodies against intracellular antigens (Table 6B, Fisher's exact test: *p* < 0.005, Bayesian BF Poisson = 3.26). However, no differences between the autoantibody subgroups with regard to the HiTOP spectra and subfactors could be demonstrated (Tables 6C,D). Furthermore, we performed an analysis of only NMDAR antibodies compared to PsychAb – PB patients as NMDAR depicted the largest size of one autoantibody subtype. We found less attention and memory impairment in patients with NMDAR autoantibodies (*n* = 3) than in PsychAb – PB patients (*n* = 96) (Supplementary Table 1A, Fisher's exact test: *p* < 0.05, Bayesian BF Poisson = 2.09). In addition, patients with NMDAR showed fewer formal thinking disorders (*n* = 2) compared to PsychAb-PB patients (*n* = 83) (Supplementary Table 1A, Fisher's exact test: S < 0.05, Bayesian BF Poisson = 2.09). After an in-depth analysis of affective psychopathology, it has been shown that patients with NMDAR autoantibodies had a greater percentage of the feeling of loss of vitality (Supplementary Table 1B, Fisher's exact test: *p* < 0.05, Bayesian BF Poisson = 0.85) and ambivalence (Supplementary Table 1B, Fisher's exact test: *p* < 0.05, Bayesian BF Poisson = 0.58) compared to PsychAb – PB patients. No relevant differences emerged between NMDAR antibody-positive patients and PsychAb – PB patients concerning HiTOP subfactors and spectra (Supplementary Tables 1C,D).

**Table 6:** (A–D) Autoantibody subgroup analysis.

**A**	**Autoantibodies against intracellular antigens**, ***n*** = **17**		**Autoantibodies against membrane surface antigens**, ***n*** = **15**		**Fischer's exact test**	**Effect size**	**Bayesian test**	**Log odds ratio**
**AMDP domains**	**Not present**	**Present**	**Not present**	**Present**	***p*-value**	**Cramers V**	**BF 10 poisson**	
**Disturbance of consciousness**	
Disturbances of orientation	35.2%	64.7%	53.3%	46.6%	0.305	0.181	1.277	0.703 (−0.664/2.070)
Disturbances of memory and attention	5.9%	94.1%	53.3%	46.7%	0.003	0.527	55.302	2.485 (0.593/4.378)
Formal thought disorders	29.4%	70.6%	53.3%	46.7%	0.169	0.243	1.882	0.944 (−0.468/2.356)
Worries and compulsions	88.2%	11.8%	80%	20%	0.522	0.113	0.702	−0.537 (−2.306/1.233)
Delusions	88.2%	11.8%	93.3%	6.7%	0.621	0.087	0.524	0.441 (−1.673/2.556)
Disorders of perception	94.1%	5.9%	86.7%	13.3%	0.471	0.128	0.599	−0.695 (−2.812/1.421)
Ego disturbances	94.1%	5.9%	93.3%	6.7%	0.927	0.016	0.42	−0.127 (−2.449/2.196)
Disturbances of affect	29.412%	70.6%	21.4%	78.5%	0.613	0.091	0.776	−0.367 (−1.913/1.179)
Disorders of drive and psychomotor activity	47.1%	52.9%	26.7%	73.3%	0.234	0.21	1.468	−0.806 (−2.214/0.602)
Circadian disturbances	94.1%	5.9%	86.7%	13.3%	0.471	0.128	0.599	−0.721 (−2.855/1.432)
Other disturbances	52.9%	47.1%	53.3%	46.7%	0.982	0.004	0.78	0.020 (−1.329/1.369)
**B**	**Autoantibodies against intracellular antigens**, ***n*** = **17**		**Autoantibodies against membrane surface antigens**, ***n*** = **15**		**Fischer's exact test**	**Effect size**	**Bayesian test**	**Log odds ratio**
**AMDP items**	**Not present**	**Present**	**Not present**	**Present**	***p*** **-value**	**Cramers V**	**BF 10 poisson**	
**Affective disturbances**								
Perplexity	94.1%	5.9%	100%	–	0.34	0.169	0.406	0.924 (−2.129/3.976)
Feeling of loss of feeling	100%	–	100%	–				
Blunted affect	76.5%	23.5%	100%	–	0.045	0.355	3.259	2.238 (−0.527/5.002)
Feeling loss of vitality	100%	–	86.7%	13.3%	0.12	0.275	1.019	−1.761 (−4.664/1.141)
Depressed mood	52.9%	47.1%	73.33%	26.7%	0.234	0.21	1.468	0.822 (−0.622/2.267)
Hopelessness	94.1%	5.9%	93.3%	6.7%	0.927	0.016	0.42	−0.129 (−2.452/2.194)
Anxiety	70.6%	29.4%	60%	40%	0.529	0.111	0.899	−0.441 (−1.861/0.978)
Euphoria	100%	–	100%	–				
Dysphoria	88.2%	11.8%	93.3%	6.7%	0.621	0.087	0.524	−0.434 (−2.534/1.667)
Irritability	94.118%	5.882%	86.667%	13.3%	0.471	0.128	0.599	0.705 (−1.439/2.849)
Inner restlessness	88.235%	11.8%	73.3%	26.7%	0.281	0.191	1.053	0.884 (−0.839/2.607)
Complaintiveness	100%	–	93.3%	6.7%	0.279	0.191	0.46	1.187 (−1.855/4.228)
Feelings of inadequacy	94.1%	5.9%	86.7%	13.3%	0.471	0.128	0.599	0.693 (1.426/2.812)
Exaggerated self-esteem	94.118%	5.9%	100%	–	0.34	0.169	0.406	0.927 (−2.109/3.964)
Feelings of guilt	100%	–	93.3%	6.7%	0.279	0.191	0.46	−1.146 (−4.129/1.836)
Feelings of impoverishment	100%	–	100%	–				
Ambivalence	100%	–	93.3%	6.7%	0.279	0.191	0.46	−1.202 (−4.302/1.898)
Parathymia	100%	–	100%	–				
Affective lability	100%	–	80%	20%	0.053	0.342	2.351	−2.165 (−4.951/0.622)
Affective incontinence	100%	–	100%5	–				
Affective rigidity	70.6%	29.4%	86.7%	21.9%	0.272	0.194	1.117	0.845 (−0.849/2.539)
**C**	**Autoantibodies against intracellular antigens**, ***n*** = **17**		**Autoantibodies against membrane surface antigens**, ***n*** = **15**		**Fischer's exact test**	**Effect size**	**Bayesian test**	**Log odds ratio**
**HiTOP spectra**	**Not present**	**Present**	**Not present**	**Present**	***p*** **-value**	**Cramers V**	**BF 10 poisson**	
Somatoform	94.2%	5.9%	93.3%	6.7%	0.927	0.016	0.42	−0.134 (−2.509/2.241)
Internalizing	64.7%	35.4%	80%	20%	0.337	0.17	1.074	0.691 (−0.826/2.208)
Thought disorder	88.2%	11.8%	80%	20%	0.522	0.113	0.702	−0.533 (−2.303/1.237)
Disinhibited externalizing	88.2%	11.8%	93.3%	6.7%	0.621	0.087	0.524	0.443 (−1.646/2.533)
Antagonistic externalizing	100%	–	100%	–	–	–	–	
Detachment	100%	–	100%	–	–	–	–	
**D**	**Autoantibodies against intracellular antigens**, ***n*** = **17**		**Autoantibodies against membrane surface antigens**, ***n*** = **15**		**Fischer's exact test**	**Effect size**	**Bayesian test**	**Log odds ratio**
**HiTOP subfactors**	**No present**	**Present**	**No present**	**Present**	***p*** **-value**	**Cramers V**	**BF 10 poisson**	
Sexual problems	100%	–	100%	–	–	–	–	
Eating pathology	100%	–	100%	–	–	–	–	
Fear	94.1%	5.9%	100%	–	0.34	0.169	0.406	−0.940 (−4.025/2.146)
Distress	64.7%	35.3%	86.7%	13.3%	0.152	0.253	1.746	−1.111 (2.760/0.538)
Mania	100%	–	93.3%	6.7%	0.279	0.191	0.46	1.175 (−1.848/4.198)
Substance abuse	94.1%	5.9%	93.3%	6.7%	0.921	0.016	0.42	0.177 (−2.213/2.447)
Antisocial behavior	94.1%	5.9%	100%	–	0.34	0.169	0.406	−0.954 (−3.982/2.075)

## Discussion

Our study revealed that orientation disorder is a specific psychopathological feature that is more prominent in neural autoantibody-associated psychiatric syndromes compared to those without antibodies. On the contrary, a blunted affect and affective rigidity appears less often in psychiatric patients with than in those without neural autoantibodies. Our results in the field of cognitive and affective psychopathology applying a statistical procedure (Fisher's exact test) were confirmed in another, different statistical procedure, namely Bayesian statistic, obtaining Bayesian values between 3 and 13 regarding the psychopathological items orientation disorder, blunted affect and affective rigidity. Another affective symptom more often present in neural autoantibody-positive psychiatric patients is a loss of vitality, if those patients presenting cerebrospinal-fluid autoantibodies are investigated in particular. Furthermore, autoantibody-positive psychiatric patients more often have a manic syndrome than those without autoantibodies, thus confirming studies showing an association between mania and autoantibodies like NMDAR antibodies (15). These results suggest that affective and cognitive dysfunction are important domains in which the psychopathology should very carefully assessed.

### Psychopathology in patients with autoantibody subgroups

Our subgroup analysis showed that patients with intracellular autoantibodies were much more likely to have attention and memory impairments compared with patients with membrane surface autoantibodies. These results are consistent with previous studies showing cognitive impairment in patients with subsets of intracellular antibodies such as recoverin antibodies, published recently (16, 17). It is likely that temporal aspects of the presence of autoantibodies and the development of cognitive dysfunction are the reason that patients with autoantibodies to intracellular antigens are more affected than those with autoantibodies to membrane surface antigens. In addition, we examined the largest group of autoantibodies to a single antigen relative to our study rather than the literature: patients with NMDAR autoantibodies (*n* = 7). Analyses of the homogenous group of patients with NMDAR antibodies compared to antibody-negative patients also showed that although there were changes in psychopathology (less memory and attention disturbances, less formal thought disorder, more ambivalence, and more feelings of loss of vitality), these cannot be regarded as meaningful due to the small number of cases.

### HiTOP classification vs. AMDP measurement of psychopathology associated with neural autoantibodies

No differing psychopathological features emerged when symptoms were analyzed *via* the HiTOP classification being in agreement with a recent controversy on HiTOP measurement system (18, 19) postulating disadvantages in HiTOP classification system in its current version for clinical use (18). The AMDP thus seems more suitable for assessing psychiatric symptoms potentially prominent in autoantibody-associated psychiatric syndromes. However, we could not confirm any relationship between neural autoantibody intensity and any main psychopathological feature in any of the measurement systems, as was recently demonstrated in terms of cognitive dysfunction in a subgroup of patients with psychiatric syndromes and associated autoantibodies—namely autoimmune psychosis—revealing a correlation between NMDAR autoantibody titer and cognitive dysfunction (20). Thus, our findings show that the AMDP measurement scale is superior to the HiTOP scale in detecting differences in the psychopathology of psychiatric patients between those with and those without autoantibodies. We believe that a detailed, single symptom-oriented approach is more fruitful than an overstretched grouping of symptom domains, as small differences might prove to be extremely helpful for differentiating disease entities (autoantibody-related vs. non-autoantibody-related).

### Specific psychopathology in autoantibody-associated psychiatric syndromes

The evidence of prominent orientation deficits has been reported in autoantibody-associated psychiatric syndromes (17, 21). The limbic system plays a key role in spatial orientation, which is often affected in the autoimmune encephalitis called limbic encephalitis, but it might be also functionally impaired in autoantibody-associated psychiatric syndromes leading to orientation deficits. The possibility of a different affective psychopathology in autoantibody-associated psychiatric syndromes should be kept in mind: A blunted affect is more pronounced in antibody-negative than antibody-positive psychiatric syndromes, underlying a somewhat maintained sensation capability in an autoantibody-associated psychiatric syndrome. The psychopathology of 103 patients with Sjögren's syndrome was assessed extensively in association with autoantibodies against neuropeptides in comparison with 110 healthy controls (22). In their study, the patients with Sjögren's syndrome and autoantibodies against neuropeptides revealed different degrees of psychopathological characteristics, indicating that assessing personality traits is a potentially promising research field to target also in conjunction with autoantibody-associated psychiatric syndromes. This point was not investigated in detail in our current study. Thus, we confirm in our study patients a specific psychopathology revealing more orientation deficits, less blunted affect, and more loss of vitality, which point toward a phenotype entailing lost energy and orientation deficits in autoantibody-associated psychiatric syndromes. Our findings with mild psychopathological abnormalities in individual AMDP items, but not major AMDP domains in psychiatric patients with autoantibodies, should be investigated in further studies with larger numbers of patients with specific autoantibody classes. Another important aspect to consider is that differences in psychopathology might not depend so much on the underlying psychiatric syndrome, as we found no differences between main syndromes apart from the manic syndrome of antibody-positive patients compared to antibody-negative patients. Thus, it is reasonable to measure a wide spectrum of psychopathologic items to be certain to detect small differences that might be of additional differential diagnostic value.

### Limitations

The heterogeneous composition of our autoantibody-positive group is a potential limitation as the psychopathology associated with specific autoantibodies might be unique. Further studies with homogeneous autoantibody groups are needed to clarify this issue. Furthermore, neural autoantibody groups should be examined in separate groups, and their psychiatric diagnoses should likewise be differentiated to discover whether certain autoantibodies play a role in specific psychiatric diseases. It would also be worthwhile to address this question in a future study with more homogeneous psychiatric-symptom constellations, and similar groups of neural autoantibodies (chosen for their supposed mechanism of action, and intra- or extracellular location of neural autoantibodies) in the hope that an immunotherapy approach would help to alleviate the symptoms of patients with a probable autoimmune basis. Another limitation is that patients with comorbidities such as other non-psychiatric disorders, developmental anomalies, or neurologic conditions were not excluded, as these are possible confounding factors. Furthermore, we cannot exclude that other factors like substance abuse or use, or anxiety might have an impact on psychopathology, not just the neural autoantibody biomarker. We thus recommend considering all these limitations in a future study to exclude confounding factors in a large cohort of autoantibody-positive psychiatric patients. Note that we refrained from multivariant testing and refer to our study's exploratory nature (we also dispensed with multivariant models because of its exploratory nature). A MANOVA is a suitable method that could be useful for a larger cohort to detect differences in psychopathology between antibody-positive and antibody-negative psychiatric patients. Another caveat is the timing of symptoms, which cannot be optimally assessed *via* a cross-sectional approach. In another study, we would prefer to assess psychopathology across its spectrum longitudinally, since in neural autoantibody diseases, symptoms often have a relapsing remitting character. A prospective study with fixed time points is necessary in future to avoid test biases regarding the timing of psychometric evaluation. Another limitation is that we only have data on the semiquantitative intensity assessment of neuronal autoantibodies and no endpoint titers of all autoantibodies, which is not a suitable biomarker to support the clinical relevance of autoantibody findings. In addition, as a further limitation of our study, it is worth noting that in the evaluation of psychopathology, the notes of doctors are used. However, this is not a prospective assessment that is carried out the same for everyone, so our study hereby draws attention to this limitation.

## Conclusion

To the best of our knowledge, this is the first study to compare two methods for assessing psychopathology in autoantibody-associated psychiatric patients; we encourage the implementation of psychometry and classification of psychiatric syndromes as an important diagnostic component in the differential diagnosis of suspected autoimmune psychiatric syndromes. Thorough psychometry should be part of the diagnostic approach if an organic cause of psychiatric symptoms is suspected, and even when no organic cause of them is expected, as a patient's psychopathology may reveal important clues to facilitate their diagnostics and care. A standard psychometric assessment scale like the AMDP seems adequate for detecting such slight differences in the phenotypical appearance of psychiatric disease associated with neural autoantibodies. The strength of our study is our detailed and novel assessment of the psychometry of patients presenting a plethora of psychopathological characteristics associated with various autoantibody-associated psychiatric syndromes. More research should be done to compare different measurement scales in assessing deep phenotypical differences between those psychiatric patients with and those without autoantibodies. The AMDP system for assessing psychopathology seems quite suitable and sensitive enough to reveal the psychopathological differences observed in psychiatric patients with and without autoantibodies, whereas the HiTOP classification system seems to be less useful for detecting autoantibody-associated differences in psychopathology. Therefore, as a general consideration of the findings, it is useful to survey the most specific psychopathology possible. Both affective psychopathology and a differential measurement of cognitive subdomains according to the AMDP system appear to be a relevant non-molecular marker for psychiatric patients diagnosed with neural autoantibodies.

## Data availability statement

The raw data supporting the conclusions of this article will be made available by the corresponding author, without undue reservation.

## Ethics statement

The studies involving human participants were reviewed and approved by Ethics Committee of the University Medical Center Göttingen. Written informed consent for participation was not required for this study in accordance with the national legislation and the institutional requirements.

## Author contributions

IG and NH wrote the manuscript. AJ and IG performed data collection. BT did part of the laboratory testing. DF and JW revised the manuscript for important intellectual content. All authors contributed to the article and approved the submitted version.

## Funding

Funding was derived from the Open access fund of the University of Göttingen. JW was supported by an Ilídio Pinho professorship, iBiMED (UIDB/04501/2020) at the University of Aveiro, Portugal.

## Conflict of interest

The authors declare that the research was conducted in the absence of any commercial or financial relationships that could be construed as a potential conflict of interest.

## Publisher's note

All claims expressed in this article are solely those of the authors and do not necessarily represent those of their affiliated organizations, or those of the publisher, the editors and the reviewers. Any product that may be evaluated in this article, or claim that may be made by its manufacturer, is not guaranteed or endorsed by the publisher.

## Supplementary material

Supplementary Figure 1 HiTOP spectra of psychiatric patients presenting serum neural autoantibodies vs. those without them. Ab+, psychiatric patients with neural autoantibodies; Ab−, psychiatric patients without neural autoantibodies; HiTOP, Hierarchical Taxonomy of Psychopathology; item+, item is present; item–, item absent; No., number.

Supplementary Figure 2 HiTOP factors of psychiatric patients presenting serum neural autoantibodies vs. those without them. Ab+, psychiatric patients with neural autoantibodies; Ab–, psychiatric patients without neural autoantibodies; HiTOP, Hierarchical Taxonomy of Psychopathology; item+, item is present; item–, item absent; No., number.

Supplementary Figure 3 Psychopathology of psychiatric patients with vs. those without neural autoantibodies—AMDP system. Ab+, psychiatric patients with neural autoantibodies; Ab–, psychiatric patients without neural autoantibodies; AMDP, Arbeitsgemeinschaft für Methodik und Dokumentation in der Psychiatrie; item+, item is present; item–, item absent; No., number.

Supplementary Figure 4 HiTOP spectra of psychiatric patients presenting serum neural autoantibodies vs. those without them Ab+, psychiatric patients with neural autoantibodies; Ab-, psychiatric patients without neural autoantibodies; HiTOP, Hierarchical Taxonomy of Psychopathology; item+, item is present; item–, item absent; No., number.

Supplementary Figure 5 HiTOP factors of psychiatric patients presenting serum neural autoantibodies vs. those without them Ab+, psychiatric patients with neural autoantibodies; Ab−, psychiatric patients without neural autoantibodies; HiTOP, Hierarchical Taxonomy of Psychopathology, item+, item is present; item–, item absent; No., number.

Supplementary Table 1 Psychopathology of patients with NMDAR autoantibodies vs. psychiatric patients without autoantibodies.

## References

[1] Graus F Titulaer MJ Balu R Benseler S Bien CG Cellucci T. A clinical approach to diagnosis of autoimmune encephalitis. Lancet Neurol. (2016) 15:391–404. 10.1016/S1474-4422(15)00401-9 26906964 PMC5066574

[2] Abboud H Probasco J Irani SR Ances B Benavides DR Bradshaw M. Autoimmune encephalitis: proposed recommendations for symptomatic and long-term management. J Neurol Neurosurg Psychiatry. (2021) 92:897–907. 10.1136/jnnp-2020-325302 33649021 PMC8292591

[3] Abboud H Probasco JC Irani S Ances B Benavides DR Bradshaw M. Autoimmune encephalitis: proposed best practice recommendations for diagnosis and acute management. J Neurol Neurosurg Psychiatry. (2021) 92:757–68. 10.1136/jnnp-2020-325300 33649022 PMC8223680

[4] Hansen N Lipp M Vogelgsang J Vukovich R Zindler T Luedecke D. Cerebrospinal Fluid Analysis in Psychiatry Consortium. Autoantibody-associated psychiatric symptoms and syndromes in adults: a narrative review and proposed diagnostic approach. Brain Behav Immun Health. (2020) 9:100154. 10.1016/j.bbih.2020.100154 34589896 PMC8474611

[5] Herken J Prüss H. Red flags: clinical signs for identifying autoimmune encephalitis in psychiatric patients. Front Psychiatry. (2017) 8:25. 10.3389/fpsyt.2017.00025 28261116 PMC5311041

[6] Rogers JP Pollak TA Blackman G David AS. Catatonia and the immune system: a review. Lancet Psychiatry. (2019) 6:620–30. 10.1016/S2215-0366(19)30190-7 31196793 PMC7185541

[7] Jernbom Falk JA Galletly C Just D Toben C Baune BT Clark SR. Autoantibody profiles associated with clinical features in psychotic disorders. Transl Psychiatry. (2021) 11:474. 10.1038/s41398-021-01596-0 34518517 PMC8438048

[8] Al-Diwani A Handel A Townsend L Pollak T Leite MI Harrison PJ. The psychopathology of NMDAR-antibody encephalitis in adults: a systematic review and phenotypic analysis of individual patient data. Lancet Psychiatry. (2019) 6:235–46. 10.1016/S2215-0366(19)30001-X 30765329 PMC6384244

[9] Broome MW Bottlender R Rösler M Stieglitz RD editors. Manual for the Assessment and Documentation of Psychopathology in Psychiatry (The ADMP System). 9the ed. Göttingen: Hofgrefe (2017).

[10] Kotov R Krueger RF Watson D Achenbach TM Althoff RR Bagby RM. The Hierarchical Taxonomy of Psychopathology (HiTOP): a dimensional alternative to traditional nosologies. J Abnorm Psychol. (2017) 126:454–77. 10.1037/abn0000258 28333488

[11] Krueger RF Kotov R Watson D Forbes MK Eaton NR Ruggero CJ. Progress in achieving quantitative classification of psychopathology. World Psychiatry. (2018) 17:282–93. 10.1002/wps.20566 30229571 PMC6172695

[12] Watson D Levin-Aspenson HF Waszczuk MA Conway CC Dalgleish T Dretsch MN. HiTOP Utility Workgroup. Validity and utility of Hierarchical Taxonomy of Psychopathology (HiTOP): III Emotional dysfunction superspectrum. World Psychiatry. (2022) 21:26–54. 10.1002/wps.20943 35015357 PMC8751579

[13] Kotov R Jonas KG Carpenter WT Dretsch MN Eaton NR Forbes MK. HiTOP Utility Workgroup. Validity and utility of Hierarchical Taxonomy of Psychopathology (HiTOP): I Psychosis superspectrum. World Psychiatry. (2020) 19:151–72. 10.1002/wps.20730 32394571 PMC7214958

[14] Pollak TA Lennox BR Müller S Benros ME Prüss H Tebartz van Elst L. Autoimmune psychosis: an international consensus on an approach to the diagnosis and management of psychosis of suspected autoimmune origin. Lancet Psychiatry. (2020) 7:93–108. 10.1016/S2215-0366(19)30290-1 31669058

[15] Pearlman DM Najjar S. Meta-analysis of the association between N-methyl-d-aspartate receptor antibodies and schizophrenia, schizoaffective disorder, bipolar disorder, and major depressive disorder. Schizophr Res. (2014) 157:249–58. 10.1016/j.schres.2014.05.001 24882425

[16] Hansen N Malchow B Zerr I Stöcker W Wiltfang J Timäus C. Neural cell-surface and intracellular autoantibodies in patients with cognitive impairment from a memory clinic cohort. J Neural Transm (Vienna). (2021) 128:357–69. 10.1007/s00702-021-02316-0 33677623 PMC7969694

[17] Hansen N Juhl AL Grenzer IM Hirschel S Teegen B Fitzner. Cerebrospinal fluid total tau protein correlates with longitudinal, progressing cognitive dysfunction in anti-neural autoantibody-associated dementia and Alzheimer's dementia: a case-control study. Front Immunol. (2022) 13:837376. 10.3389/fimmu.2022.837376 35309366 PMC8927820

[18] Haeffel GJ Jeronimus BF Kaiser BN Weaver LJ Soyster PD Fisher AJ. Folk classification and factor rotations: whales, sharks, and the problems with the Hierarchical Taxonomy of Psychopathology (HiTOP). Clin Psychol Sci. (2022) 10:259–78. 10.1177/21677026211002500 35425668 PMC9004619

[19] DeYoung CG Kotov R Krueger RF Cicero DC Conway CC Eaton NR. Answering questions about the Hierarchical Taxonomy of Psychopathology (HiTOP): analogies to whales and sharks miss the boat. Clin Psychol Sci. (2022) 10:279–84. 10.1177/21677026211049390 35444863 PMC9017579

[20] Abe K Chiba Y Katsuse O Takahashi Y Suda A Hattori S. Exploratory investigation on antibodies to GluN1 and cognitive dysfunction in patients with chronic autoimmune psychosis. Neurosci Lett. (2021) 743:135588. 10.1016/j.neulet.2020.135588 33359543

[21] Endres D Lüngen E Hasan A Kluge M Fröhlich S Lewerenz J. Clinical manifestations and immunomodulatory treatment experiences in psychiatric patients with suspected autoimmune encephalitis: a case series of 91 patients from Germany. Mol Psychiatry. (2022) 27:1479–89. 10.1038/s41380-021-01396-4 35046526 PMC9095476

[22] Karaiskos D Mavragani CP Sinno MH Déchelotte P Zintzaras E Skopouli FN. Psychopathological and personality features in primary Sjogren's syndrome–associations with autoantibodies to neuropeptides. Rheumatology. (2010) 49:1762–9. 10.1093/rheumatology/keq158 20525741

