# Supplementary figures and images for: Psychopathology of psychiatric patients presenting autoantibodies against neuroglial antigens
Source: Front Psychiatry. 2022 Nov 10;13:945549. doi: 10.3389/fpsyt.2022.945549 (PMC9685427; doi:10.3389/fpsyt.2022.945549)

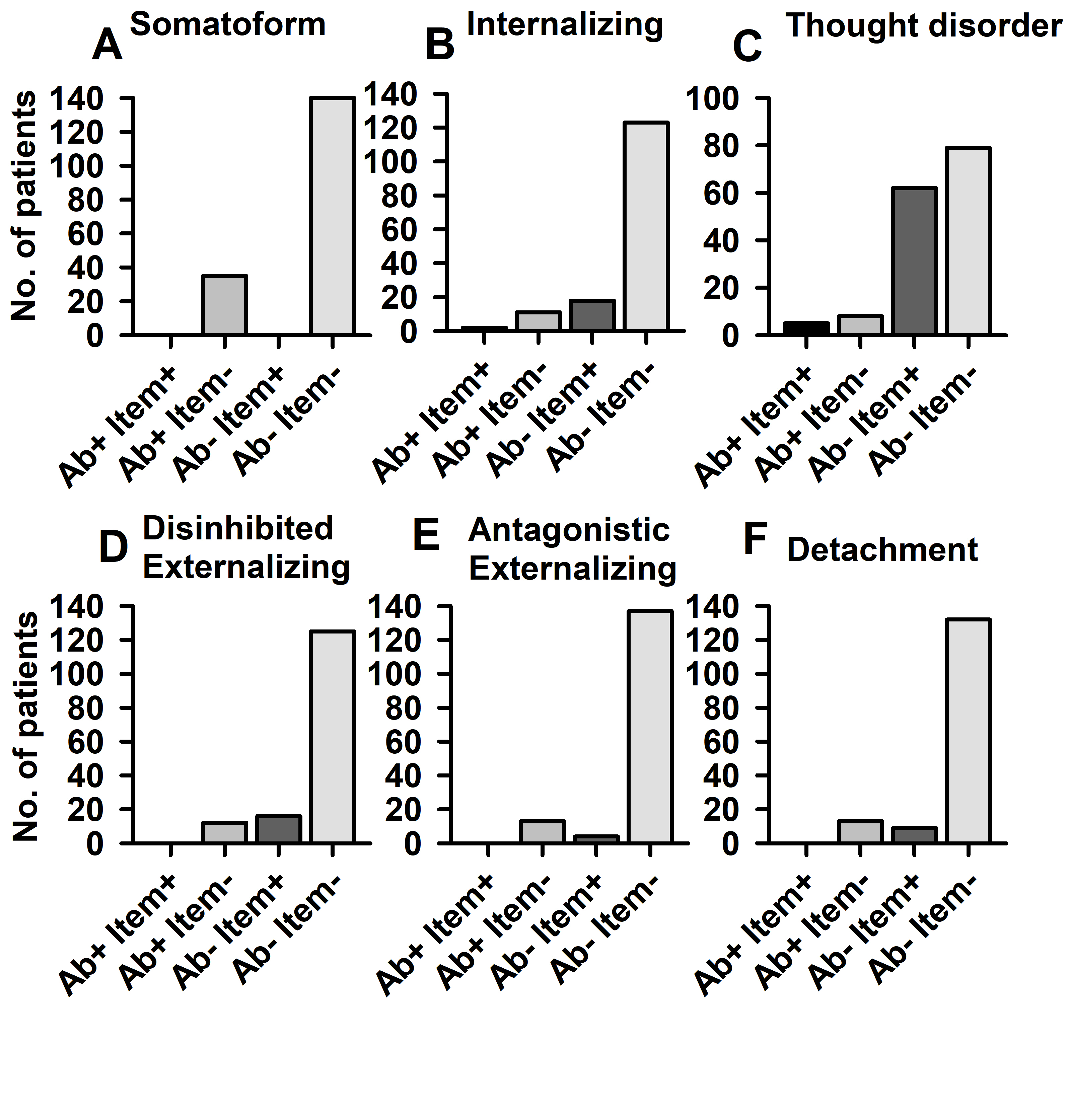

Supplement: Supplementary Figure 1 — HiTOP spectra of psychiatric patients presenting serum neural autoantibodies vs. those without them. Ab+, psychiatric patients with neural autoantibodies; Ab−, psychiatric patients without neural autoantibodies; HiTOP, Hierarchical Taxonomy of Psychopathology; item+, item is present; item–, item absent; No., number. [file Image_1.JPEG]

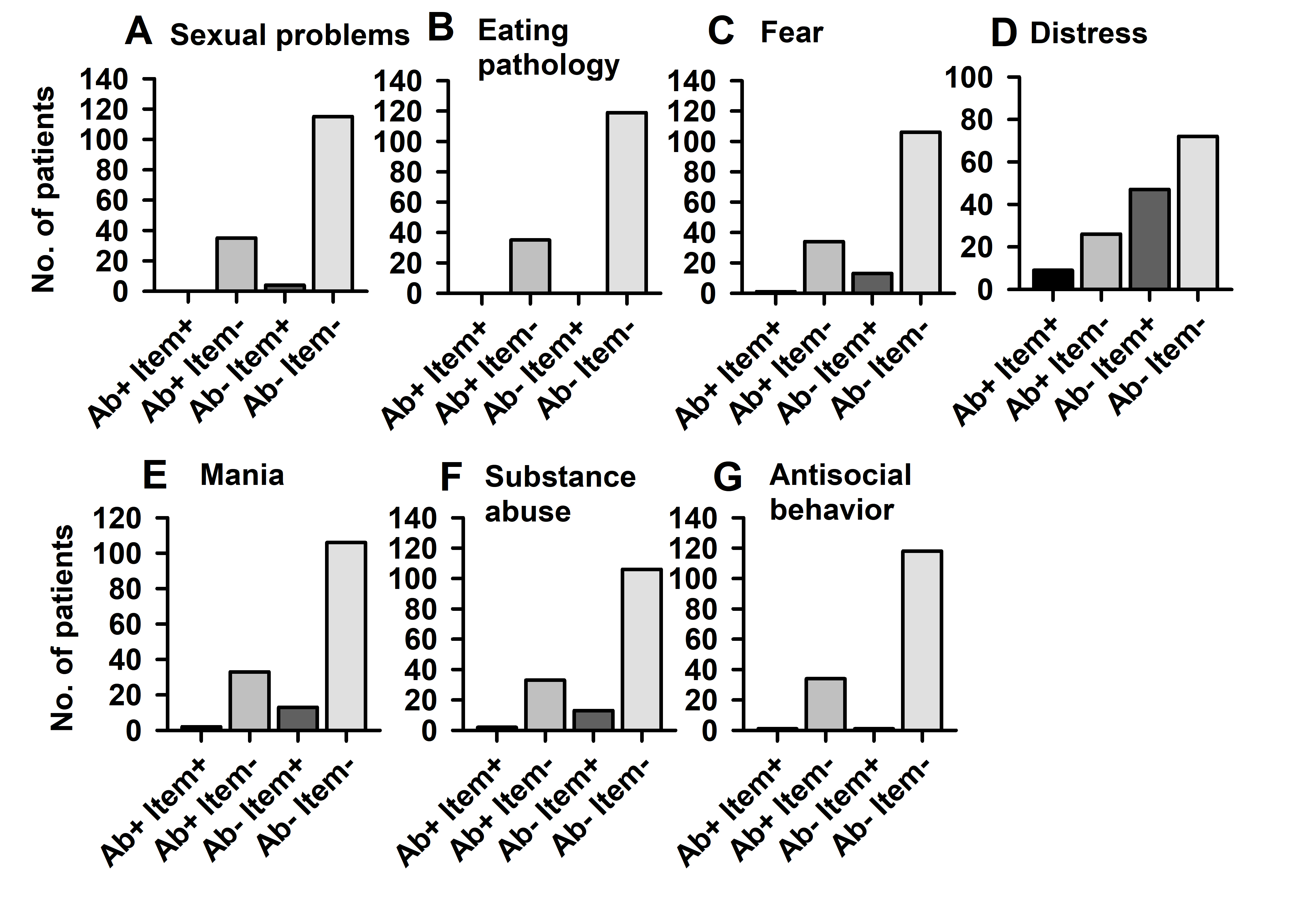

Supplement: Supplementary Figure 2 — HiTOP factors of psychiatric patients presenting serum neural autoantibodies vs. those without them. Ab+, psychiatric patients with neural autoantibodies; Ab–, psychiatric patients without neural autoantibodies; HiTOP, Hierarchical Taxonomy of Psychopathology; item+, item is present; item–, item absent; No., number. [file Image_2.JPEG]

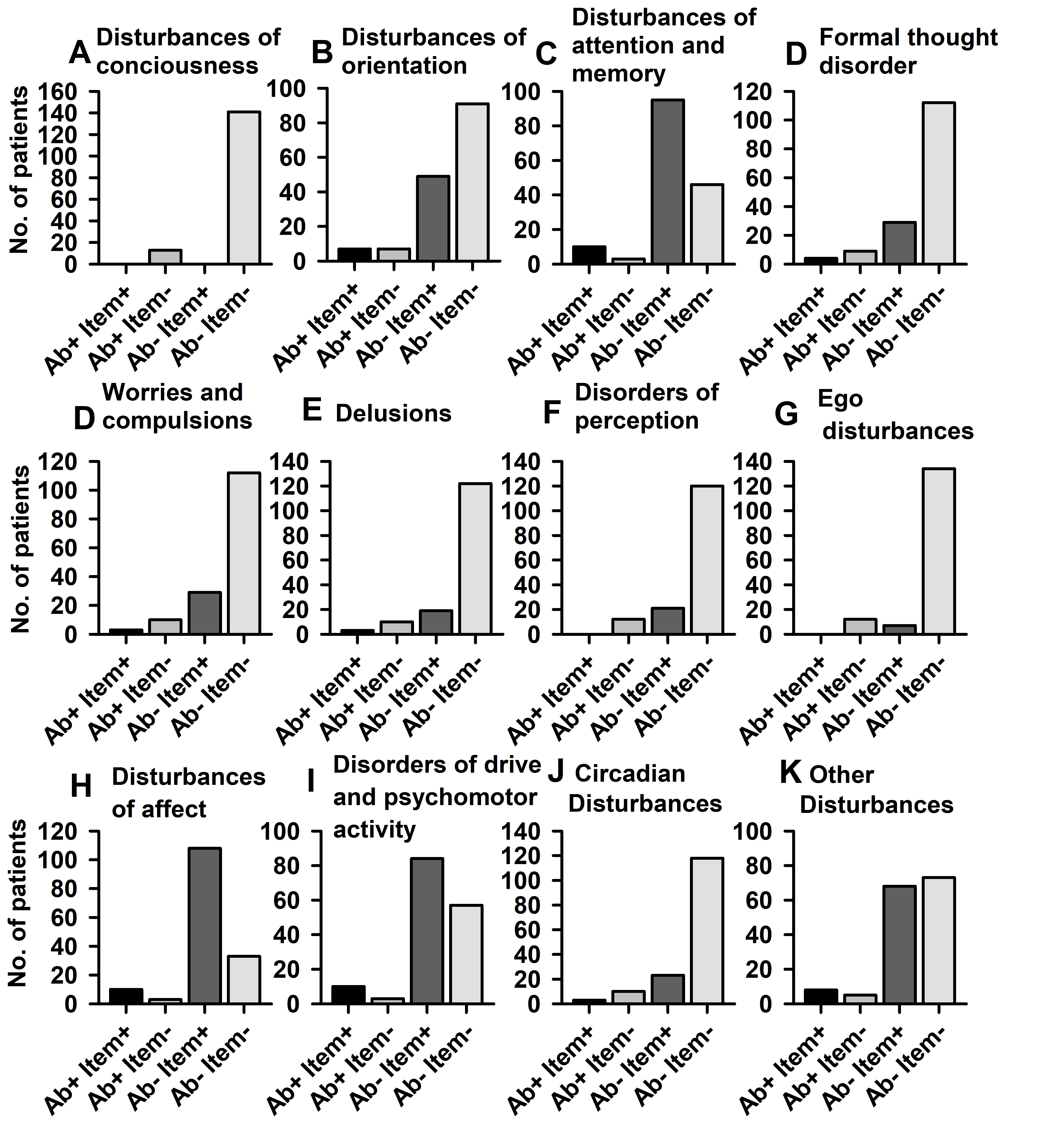

Supplement: Supplementary Figure 3 — Psychopathology of psychiatric patients with vs. those without neural autoantibodies—AMDP system. Ab+, psychiatric patients with neural autoantibodies; Ab–, psychiatric patients without neural autoantibodies; AMDP, Arbeitsgemeinschaft für Methodik und Dokumentation in der Psychiatrie; item+, item is present; item–, item absent; No., number. [file Image_3.JPEG]

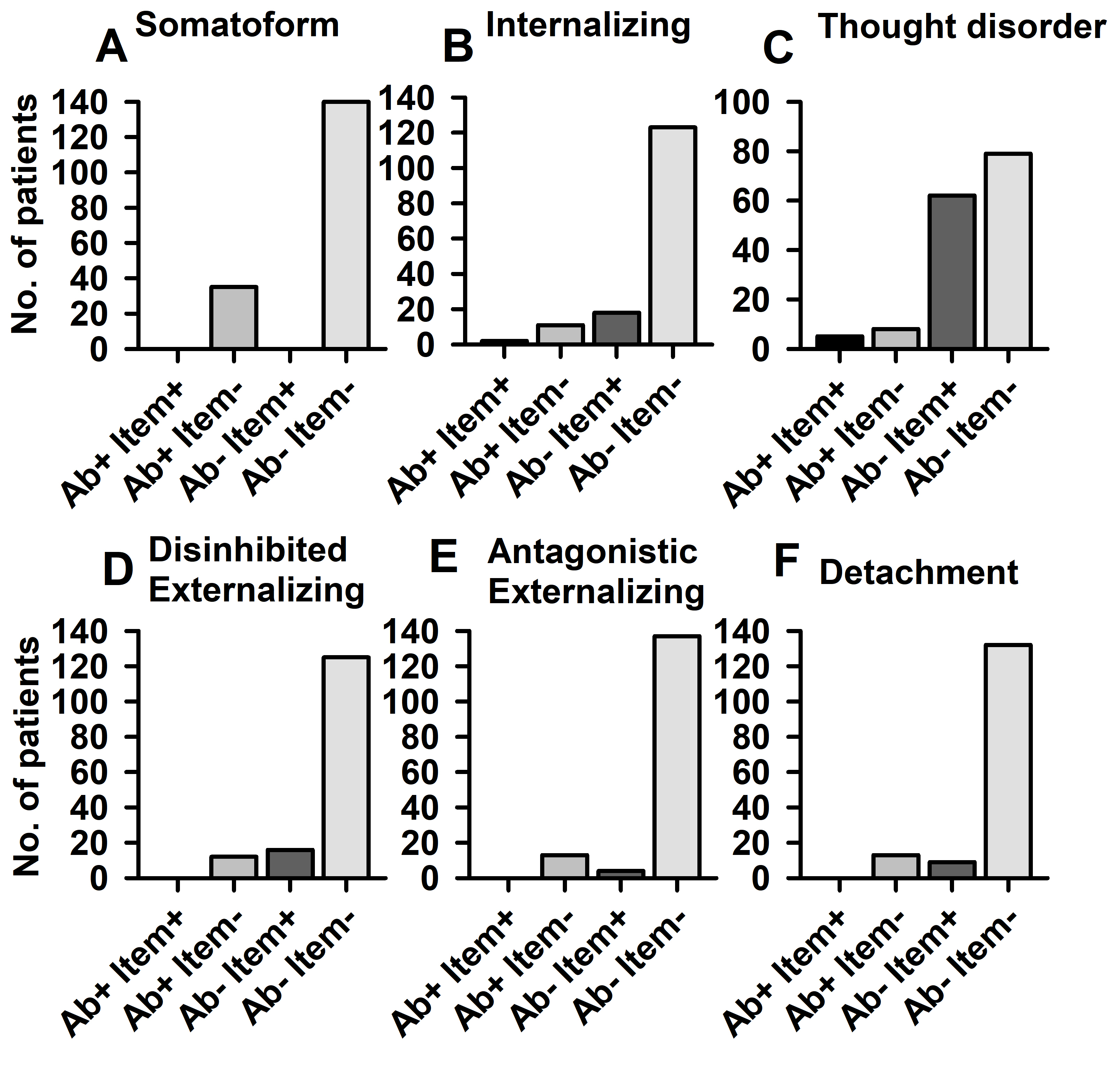

Supplement: Supplementary Figure 4 — HiTOP spectra of psychiatric patients presenting serum neural autoantibodies vs. those without them Ab+, psychiatric patients with neural autoantibodies; Ab-, psychiatric patients without neural autoantibodies; HiTOP, Hierarchical Taxonomy of Psychopathology; item+, item is present; item–, item absent; No., number. [file Image_4.JPEG]

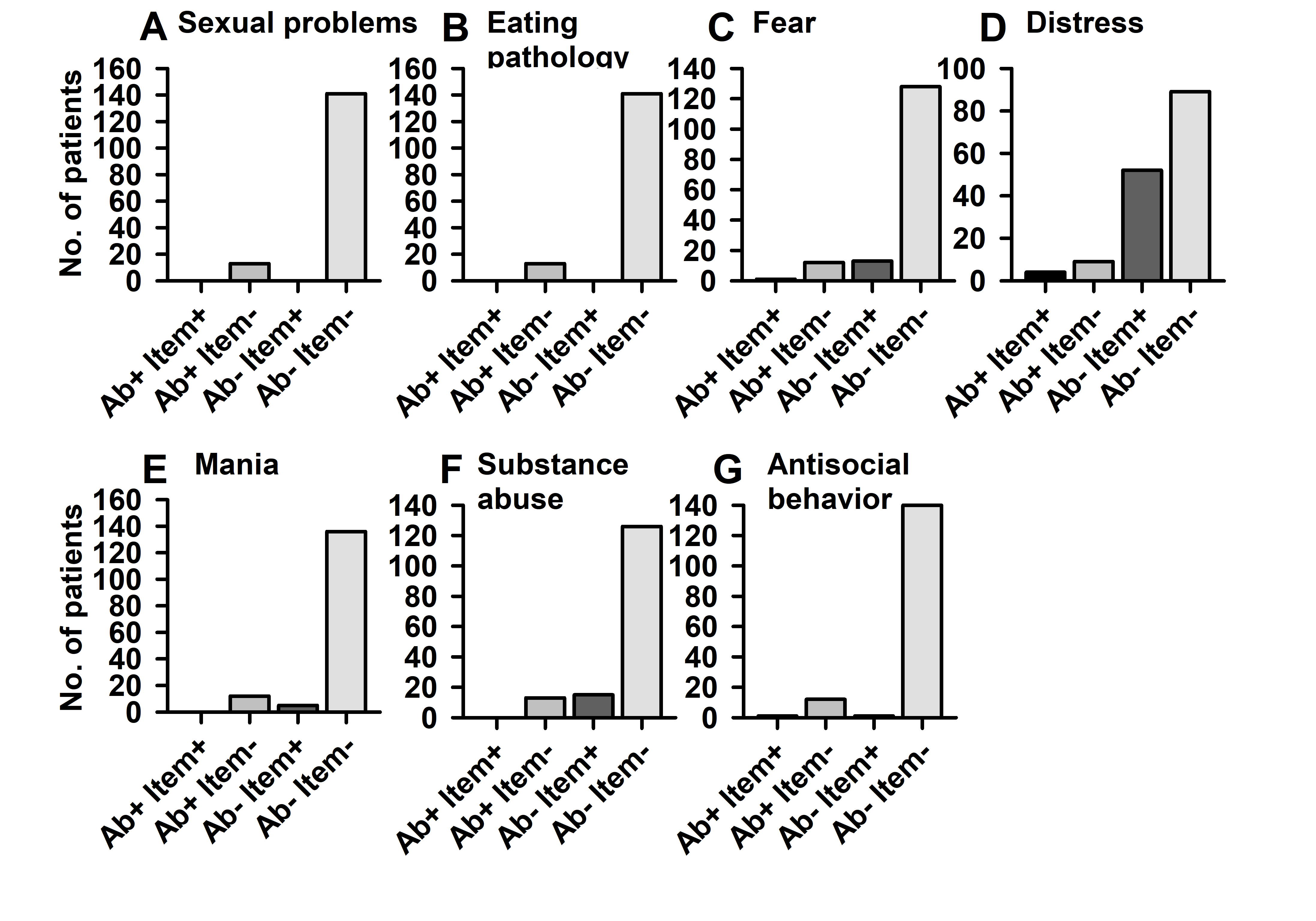

Supplement: Supplementary Figure 5 — HiTOP factors of psychiatric patients presenting serum neural autoantibodies vs. those without them Ab+, psychiatric patients with neural autoantibodies; Ab−, psychiatric patients without neural autoantibodies; HiTOP, Hierarchical Taxonomy of Psychopathology, item+, item is present; item–, item absent; No., number. [file Image_5.JPEG]
