# Supplementary material for: Psychopathology of psychiatric patients presenting autoantibodies against neuroglial antigens
Source: Front Psychiatry. 2022 Nov 10;13:945549. doi: 10.3389/fpsyt.2022.945549 (PMC9685427; doi:10.3389/fpsyt.2022.945549)
Supplement: Supplementary Table 1 — Psychopathology of patients with NMDAR autoantibodies vs. psychiatric patients without autoantibodies. [file Table_1.pdf]

Supplement Table 1: Psychopathology of patients with NMDAR autoantibodies versus psychiatric patients without autoantibodies

A	Anti- NMDAR autoantibodies n=7		Autoantibody negative n=118		Fischer's exact test	Effect size	Bayesian Test	Log Odds Ratio
AMDP domains	not present	present	not present	present	p-value	Cramers V	BF 10 Poisson	
Disturbance of consciousness	100%		100%					
Disturbances of orientation	42,9%	57,1%	67,8%	32,2%	0,175	0,121	0,477	-0,986 (-2,420/ 0,448)
Disturbances of memory and attention	57,1%	42,9%	18,6%	81,4%	0,015	0,218	2,092	1,713 (0,249/3,176)
Formal thought disorders	71,4%	28,6%	29,7%	70,3%	0,021	0,206	2,089	1,643 (0,127/ 3,159)
Worries and compulsions	71,4%	28,5%	77,1%	22,9%	0,729	0,031	0,198	-0,423 (-1,959/ 1,113)
Delusions	100%	.	83,9%	16,1%	0,249	0,103	0,201	0,963 (-1,661/ 3,587)
Disorders of perception	85,7%	14,3%	83,9%	16,1%	0,899	0,011	0,152	-0,171 (-1,991/ 1,649)
Ego disturbances	100%	.	94,9%	5,1%	0,541	0,055	0,088	-0,268 (-3,013/ 2,477)
Disturbances of affect	14,3%	85,7%	22,0%	77,9%	0,628	0,043	0,173	-0,222 (-2,047/ 1,603)
Disorders of drive and psychomotor activity	28,6%	71,4%	39,8%	60,2%	0,553	0,053	0,226	-0,390 (-1,929/ 1,149)
Circadian disturbances	85,7%	14,3%	81,4%	18,6%	0,772	0,026	0,158	-0,003 (-1,827/ 1,820)
Other disturbances	57,1%	42,9%	51,7%	48,3%	0,779	0,025	0,212	0,186 (-1,259/ 1,632)

Supplement Table 1: Psychopathology of patients with NMDAR autoantibodies versus psychiatric patients without autoantibodies

B	Anti- NMDAR autoantibodies n=7		Autoantibody negative n=118		Fischer's exact test	Effect size	Bayesian Test	Log Odds Ratio
AMDP items Affectivity disturbances	not present	present	not present	present	p-value	Cramers V	BF 10 Poisson	
Perplexity	100%		93,2%	5,9%	0,505	0,06	0,094	-0,121 (-2,804/ 2,561)
Feeling of loss of feeling	100%		94,1%	5,9%	0,507	0,059	0,093	-0,116 (-2,826/ 2,594)
Blunted affect	100%		66,9%	33,1%	0,067	0,164	0,913	1,897 (-0,761/ 4,554)
Feeling loss of vitality	71,4%	28,6%	94,1%	5,9%	0,024	0,201	0,85	-1,904 (-3,566/ -0,242)
Depressed mood	71,4%	28,6%	47,5%	52,5%	0,218	0,11	0,409	0,875 (-0,650/ 2,40)
Hopelessness	85,7%	14,3%	83,0%	19,9%	0,855	0,016	0,154	-0,120 (-1,926/ 1,687)
Anxiety	57,1%	42,9%	60,2%	39,8%	0,874	0,014	0,206	-0,160 (-1,608/ 1,287)
Euphoria	100%		96,6%	3,3%	0,621	0,044	0,078	-0,644 (-3,388/ 2,099)
Dysphoria	85,7%	14,3%	93,2%	5,9%	0,455	0,067	0,184	-1,115 (-3,006/ 0,777)
Irritability	85,7%	14,3%	96,6%	3,4%	0,153	0,128	0,269	-1,796 (-3,789/ 0,197)
Inner restlessness	71,4%	28,6%	85,6%	14,4%	0,31	0,091	0,283	-0,981 (-2,544/ 0,582)
Complaintiveness	85,7%	14,3%	97,5%	2,5%	0,086	0,153	0,319	-2,062 (-4,114/ 0,010)
Feelings of inadequacy	100%		91,5%	8,5%	0,422	0,072	0,122	0,250 (-2,436/ 2,936)
Exaggerated self-esteem	100%		100%					
Feelings of guilt	100%		92,4%	7,6%	0,448	0,068	0,105	0,145 (-2,523/ 2,813)
Feelings of impoverishment	100%		98,3%	1,7%	0,728	0,031	0,069	-1,227 (-4,140/ 1,685)
Ambivalence	85,7%	14,3%	99,1%	0,9%	0,006	0,246	0,577	-2,899 (-5,272/ -0,525)
Parathymia	100%		98,3%	1,7%	0,806	0,022	0,066	-1,736 (-4,804/ 1,333)
Affective lability	71,5%	28,6%	89,8%	10,2%	0,134	0,134	0,421	-1,362 (-2,932/ 0,208)
Affective incontinence	100%		100%					
Affective rigidity	85,7%	14,3%	55,1%	44,9%	0,112	0,142	0,634	1,242 (-0,557/ 3,042)

Supplement Table 1: Psychopathology of patients with NMDAR autoantibodies versus psychiatric patients without autoantibodies

C	Anti- NMDAR autoantibodies n=7		Autoantibody negative n=118		Fischer's exact test	Effect size	Bayesian Test	Log Odds Ratio
HiTOP Spectra	no present	present	no present	present	p-value	Cramers V	BF 10 Poisson	
Somatoform	85,7%	14,3%	85,6%	14,4%	0,993	7,93E-04	0,151	-0,313 (-2,122/ 1,496)
Internalizing	85,7%	14,3%	53,4%	46,6%	0,095	0,149	0,731	1,321 (-0,482/ 3,124)
Thought Disorder	85,7%	14,3%	78,8%	21,2%	0,662	0,039	0,168	0,158 (-1,675/ 1,991)
Disinhibited externalizing	100%		88,2%	11,9%	0,334	0,086	0,144	0,639 (-2,067/3,345)
Antagonistic externalizing	100%		96,6%	3,4%	0,621	0,044	0,078	-0,644 (-3,437/ 2,150)
Detachment	100%		92,4%	7,6%	0,448	0,068	0,105	0,130 (-2,564/ 2,823)

D	Anti- NMDAR autoantibodies n=7		Autoantibody negative n=118		Fischer's exact test	Effect size	Bayesian Test	Log Odds Ratio
HiTOP Factors	no present	presnt	no present	present	p-value	Cramers V	BF 10 Poisson	
Sexual Problems	100%		96,6%	3,4%	0,668	0,039	0,074	-0,911 (-3,701/ 1,880)
Eating Pathology	100%		100%					
Fear	100%		89,8%	10,2%	0,375	0,079	0,127	0,456 (-2,233/ 3,144)
Distress	85,7%	14,286%	60,2%	39,8%	0,185	0,119	0,419	1,006 (-0,801/ 2,813)
Mania	100%		96,6%	3,4%	0,668	0,039	0,078	-0,640 (-3,430/ 2,149)
Substance Abuse	100%		88,9%	11,0%	0,354	0,083	0,135	0,534 (-2,143/ 3,211)
Antisocial Behavior	100%		99,2%	0,85%	0,807	0,022	0,065	-1,763 (-4,844/ 1,319)

Abbreviations:AMDP = Arbeitsgemeinschaft für Methodik und Dokumentation in der Psychiatrie, HiTOP = HIerachical Taxonomy of Psychopathology. NMDA = N-methyl-D-aspartate receptor
